# Histone lactylation promotes malignant progression by facilitating USP39 expression to target PI3K/AKT/HIF-1α signal pathway in endometrial carcinoma

**DOI:** 10.1038/s41420-024-01898-4

**Published:** 2024-03-08

**Authors:** Sitian Wei, Jun Zhang, Rong Zhao, Rui Shi, Lanfen An, Zhicheng Yu, Qi Zhang, Jiarui Zhang, Yuwei Yao, Haojia Li, Hongbo Wang

**Affiliations:** Department of Obstetrics and Gynecology, Union Hospital, Tongji Medical College, Huazhong University of Science and Technology, Wuhan, Hubei PR China

**Keywords:** Endometrial cancer, Ubiquitylation

## Abstract

Histone lactylation has been reported to involve in tumorigenesis and development. However, its biological regulatory mechanism in endometrial carcinoma (EC) is yet to be reported in detail. In the present study, we evaluated the modification levels of global lactylation in EC tissues by immunohistochemistry and western blot, and it was elevated. The non-metabolizable glucose analog 2-deoxy-d-glucose (2-DG) and oxamate treatment could decrease the level of lactylation so as to inhibit the proliferation and migration ability, induce apoptosis significantly, and arrest the cell cycle of EC cells. Mechanically, histone lactylation stimulated USP39 expression to promote tumor progression. Moreover, USP39 activated PI3K/AKT/HIF-1α signaling pathway via interacting with and stabilizing PGK1 to stimulate glycolysis. The results of present study suggest that histone lactylation plays an important role in the progression of EC by promoting the malignant biological behavior of EC cells, thus providing insights into potential therapeutic strategies for endometrial cancer.

## Introduction

Endometrial carcinoma (EC) is one of the most common tumors of the female reproductive system and is the sixth most common cancer in women [1]. EC is a type of malignant epithelial tumor that occurs in the endometrium; however, its specific pathogenesis remains unclear. Recently, the incidence of EC has increased while the age of onset has decreased [1]. Most patients are diagnosed early and have a good prognosis [2]. However, despite the increasing treatment options [3, 4], the outcomes of advanced or poorly differentiated EC and those of particular types of this malignancy are still unsatisfactory [5]. Therefore, there is an urgent need to explore the pathogenesis and effective therapeutic targets for EC in order to improve the prognosis of patients.

Epigenetic modifications play a significant role in the occurrence, development, and metastasis of tumors [6]. These modifications do not change the DNA sequence and are characterized by inheritability and reversibility [7]. Recent studies have resulted in important discoveries regarding post-translational modifications (PTMs) of histone [8]. Histone PTMs change the chromatin structure by regulating DNA-dependent processes, including transcription, replication, and DNA repair, and then regulate gene transcription and expression to play vital roles in maintaining cell homeostasis [9, 10]. These modifications function by changing the contact between nucleosomes or by recruiting nonhistones. At present, many types of histone PTMs have been identified, including acetylation [11], methylation [12], and newly discovered PTMs, such as succinylation [13], glutarylation [14], and crotonylation [15]. In-depth research has greatly expanded the understanding of the relationships between histone modifications and biological processes [16].

Most tumor cells exhibit active glucose uptake and glycolysis under aerobic conditions [17], known as the Warburg effect [18]. The Warburg effect produces a large amount of lactate, which is widely considered a metabolic byproduct. Since Zhao et al. identified histone lactylation in 2019 [19] as a new epigenetic modification that could directly stimulate gene transcription in the chromatin, an increasing number of studies have found that histone lactylation is involved in the occurrence of tumors, such as ocular melanoma [20] and liver cancer [21], as well as in the development of lung cancer [22] and renal cell carcinoma [23]. Some studies have shown that endometrial cancer tissue contains more lactate than normal endometrial tissue [24, 25]. Therefore, exploring whether histone lactate plays a role in the development of EC may lead to a new strategy for future treatment.

In this study, we found, for the first time, that histone lactylation levels were increased in EC while the suppression of histone lactylation significantly inhibited tumor progression. Mechanistically, histone lactylation promoted the expression of ubiquitin-specific peptidase 39 (USP39), which interacted with, stabilized, and de-ubiquitinated phosphoglycerate kinase 1 (PGK1), thereby activating the PI3K/AKT/HIF-1α signaling pathway. Our data reveal a novel mechanism of histone lactylation in EC tumorigenesis and provide a theoretical basis for the prevention and treatment of endometrial cancer.

## Results

### Histone lactylation levels are elevated in patients with EC

**Fig. 1:**
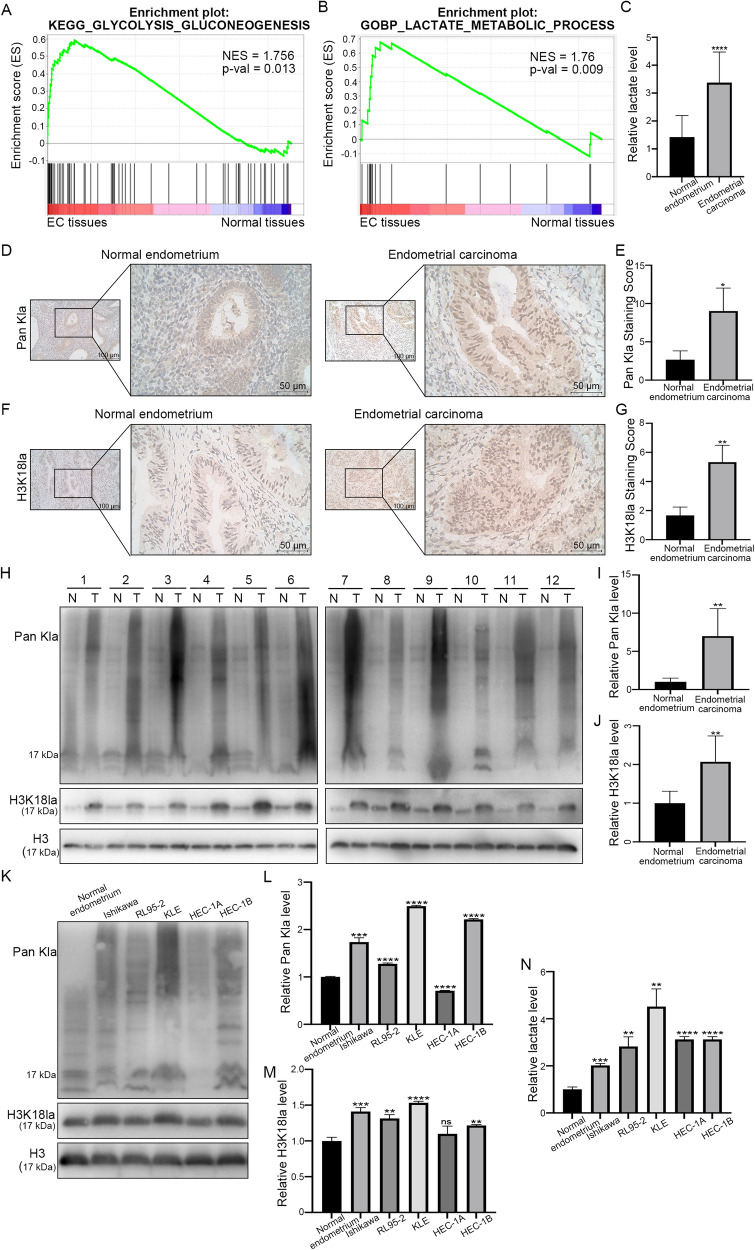
Endometrial carcinoma exhibits increased lactylation levels. **A** KEGG analysis showed EC was active in glycolysis. **B** GO analysis showed EC was enriched in the lactate metabolic process. **C** The lactate levels in EC tissues and normal endometrium were tested. **D** Representative immunohistochemistry (IHC) images of lactylation in EC and adjacent normal tissues. Scale bar: left panel, 100 μm; right panel, 50 μm. **E** The modification level of lactylation in tumor and adjacent normal tissues from EC patients was detected by IHC. **F** IHC images of H3K18la in EC tissues and adjacent normal tissues. Scale bar: left panel, 100 μm; right panel, 50 μm. **G** The modification level of H3K18la in tumor and adjacent normal tissues from EC patients was detected by IHC. **H** Lactylation and H3K18la modification levels in EC tissues and adjacent normal tissues were analyzed by western blot. **I**, **J** Densitometric analysis was performed to quantify and statistically compare lactylation and H3K18la levels in normal and EC tissues. **K** Total lactylation (Pan Kla) and H3K18la modification levels in normal endometrial cells and five EC cell lines were analyzed by western blot. **L**, **M** The lactylation and H3K18la levels in normal endometrial cells and five EC cell lines were visualized. **N** The lactate levels in normal endometrial cells and five EC cell lines were tested.

As EC shows active glycolysis and lactate metabolism (Fig. 1A, B), which may lead to a large amount of lactate production as a substrate for histone lactylation, we first determined the lactate levels in EC and normal endometrial tissues (Fig. 1C). The results showed that the lactate level in the EC tissue was significantly higher than that in normal endometrial tissue. Next, we examined the overall histone lactylation levels in EC. IHC revealed that the global lactylation level in EC tissue was dramatically elevated compared with that in normal endometrial tissue (*P* < 0.05; Fig. 1D, E). Consistently, western blotting analysis confirmed that the global histone lactylation level in EC tissues was higher than that in adjacent tissues (Fig. 1H, I). Though 28 lactylation sites were identified on core histones, only five antibodies of lactylation sites were developed (H3K18la, H3K27la, H3K14la, H3K9la, and H3K56la) by now. We detected the modification levels of these five sites between EC tissues and normal endometrial tissues and found that H3K18la was changed most significantly (Fig. S1A, B). Therefore, we investigated whether histone lactylation was caused by H3K18la in EC. Similarly, IHC showed that compared with that in normal endometrial tissue, the H3K18la level presented the same trend as the global lactylation level in EC tissue (Fig. 1F, G). Western blotting analysis also confirmed increased H3K18la levels in EC tissues compared with those in adjacent tissues (Fig. 1H, J). Next, we tested the modification levels of total lactylation and H3K18la by western blot and lactate levels by lactate detection assay in normal endometrial cells and five different EC cell lines (Fig. 1K–N). The data suggested that the level of histone lactylation was elevated in EC, which might affect EC progression. Ishikawa and KLE cells were chosen for further studies.

### Inhibition of histone lactylation suppresses proliferation and migration, induces apoptosis, and arrests cell cycle progression of EC cells in vitro

**Fig. 2:**
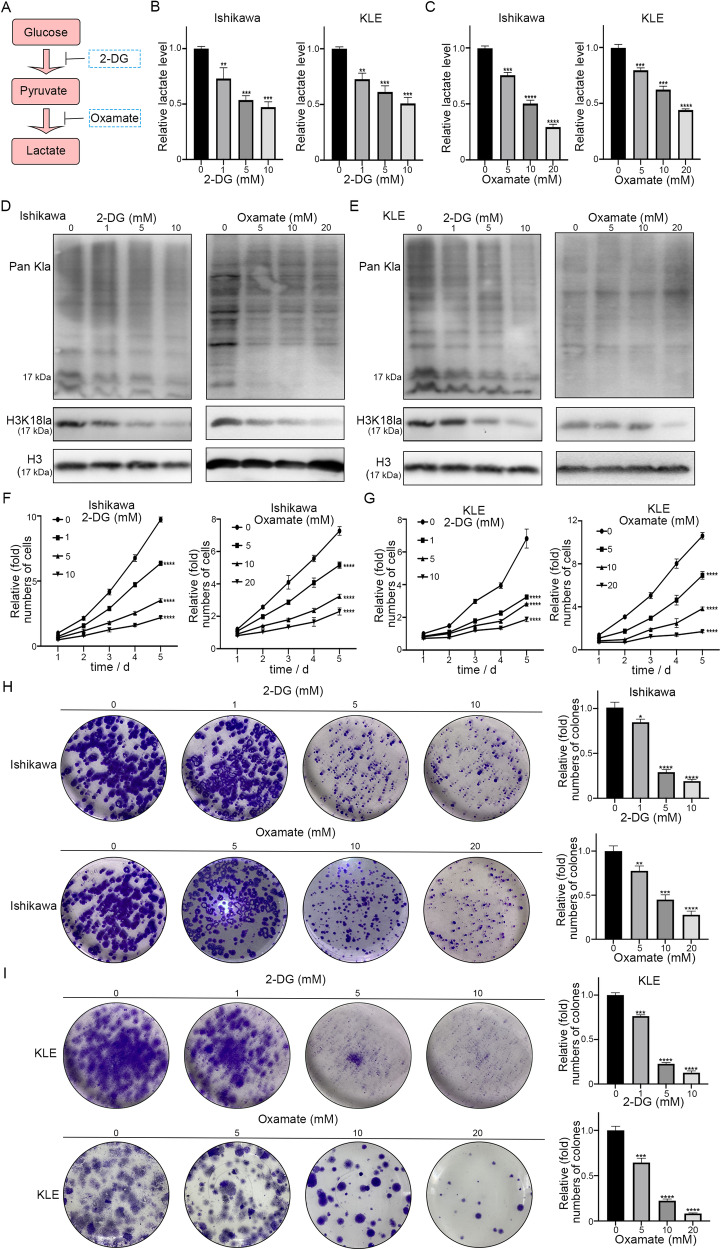
2-DG and oxamate reduces lactyation levels, and inhibition of histone lactylation suppresses EC proliferation. **A** Schematic diagram of target for inhibiting histone lactylation. **B**, **C** Intracellular lactate levels were measured from Ishikawa and KLE cells cultured in different concentrations of 2-DG or oxamate. **D**, **E** Histone lactyation and H3K18la levels were detected in Ishikawa and KLE cells cultured in different concentrations of 2-DG or oxamate by western blot. **F**, **G** Cell proliferation was detected by CCK8 assay following 2-DG or oxamate treatment in EC cells. **H**, **I** Cell proliferation was detected by colony formation assay following 2-DG or oxamate treatment in EC cells.

To investigate the functional role of histone lactylation in EC cells, we reduced the global intracellular histone lactylation levels in tumor cells. As the endogenous production of lactate is a key determinant of histone lactylation levels [19] and the non-metabolizable glucose analog (2-DG) and oxamate have been reported to be glycolysis inhibitors (Fig. 2A) [26], we adopted these compounds to attenuate lactate production and histone lactylation. The CCK-8 assay was used to determine the half-maximal inhibitory concentration (IC50) values of 2-DG and oxamate against Ishikawa and KLE cells (Fig. S2A, B). The data showed that the IC50 values of 2-DG and oxamate against Ishikawa cells were 10.22 and 17.84 mM, respectively, and those against KLE were 9.33 and 16.25 mM, respectively. Accordingly, we established corresponding concentration gradients and found that with the increases in 2-DG treatment concentrations, the relative intracellular pyruvate and lactate levels in both EC cell lines showed significant dose-dependent decreases (Fig. S2C, Fig. 2B). The same lactate content tendency was observed when treated with oxamate (Fig. 2C). Global lactylation and H3K18la levels significantly decreased in EC cells in a dose-dependent manner (Fig. 2D, E). Meanwhile, the CCK-8 assay and colony formation assays suggested that the reduction in histone lactylation effectively inhibited the proliferation of EC cells (Fig. 2F–I). Transwell assays showed that the migratory ability of EC cells was reduced after 2-DG or oxamate treatment (Fig. S3A, B). Moreover, we observed an abrupt increase in the proportion of apoptotic cells in the 2-DG-treated group compared with that in the control group (Fig. S3C). Next, we performed FACS of Ishikawa and KLE cells for cell cycle analysis. The proportions of cells in the G0/G1 phase sharply increased, whereas those in the S phase dramatically decreased after treatment with 2-DG (Fig. S3D). In summary, these findings confirm that suppressing histone lactylation can efficiently inhibit the proliferation and migration, induce apoptosis, and arrest cell cycle progression from the G0/G1 to the S phase in EC cells.

### H3K18la regulates the expression of USP39 in EC cells

**Fig. 3:**
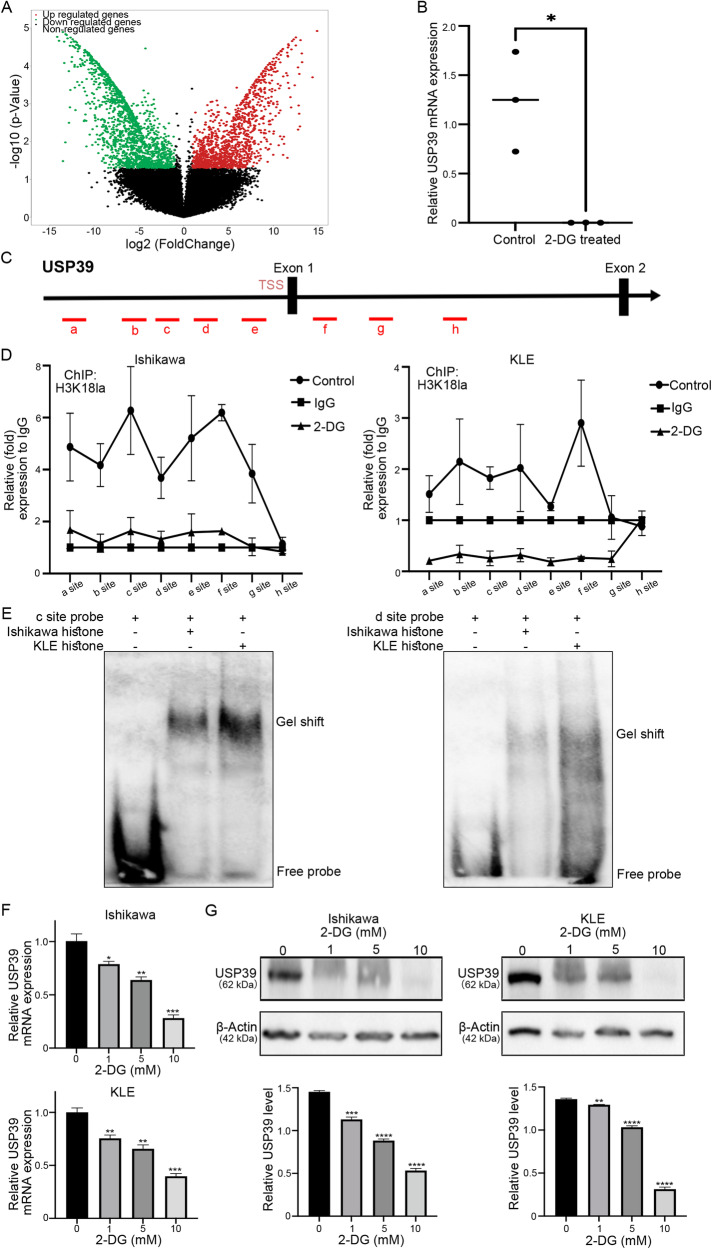
Histone lactyation regulated the expression of USP39. **A** The volcano plot showed differentially expressed genes from the RNA-seq results of Ishikawa cells treated with 2-DG or not. **B** The relative expression of USP39 mRNA from the RNA-seq results of Ishikawa cells treated with 2-DG or not. **C** Distribution of H3K18la sites relative to the translation start site (TSS) of USP39. **D** The ChIP-qPCR analysis about H3K18la enrichment in the USP39 promoter region. H3K18la enriched around the USP39 TSS, and this enrichment was reduced efficiently by 2-DG. **E** EMSA was performed to analyze the binding of histone to the USP39 promoter fragment. **F** qRT-PCR was performed to test USP39 mRNA expression in Ishikawa and KLE cells after being treated with different concentrations of 2-DG. **G** Western blot was performed to test the USP39 protein level in Ishikawa and KLE cells after being treated with varying concentrations of 2-DG.

To investigate the regulatory role of histone lactylation in gene expression, we first performed RNA sequencing (RNA-seq) of Ishikawa cells with and without 2-DG treatment (Fig. 3A). The differentially expressed genes were analyzed by GO function enrichment and KEGG pathway enrichment, and the enrichment results were visualized to understand the biological functions of the Kla-mediated molecular mechanism (Fig. S4A, B). When analyzing the RNA-seq data of the transcriptome (Table S2), we focused on ubiquitin-specific peptidase 39 (USP39), which had a significantly decreased mRNA level in 2-DG-treated cells (Fig. 3B) and has been reported to act as an oncogene in several tumors. Therefore, USP39 might be the potential downstream target of histone lactylation. To confirm that H3K18la regulated the expression of USP39, we performed ChIP-qPCR analysis, which showed that H3K18la was enriched in the USP39 promoter region (Fig. 3C, D). This enrichment was efficiently reduced by the glycolysis inhibitor 2-DG (Fig. 3C, D). To further examine whether histone could bind to the USP39 promoter active region in vitro, an EMSA was conducted. According to ChIP-PCR results, we designed biotin-labeled probes based on binding sites c (GGAGCAGCCCTGAAAGGTTTA) and d (TAAGGCTTGATGCCACACCA). After the addition of Ishikawa or KLE histone, the slow shift band appeared (Fig. 3E). These results indicated that histone indeed bound to the predicted promoter active region binding site of USP39 in vitro. As expected, the mRNA (Fig. 3F) and protein (Fig. 3G) expression levels of USP39 dramatically decreased after 2-DG treatment. Overall, these data suggest that H3K18la regulates USP39 expression in EC cells.

### USP39 is involved in the progression of EC

**Fig. 4:**
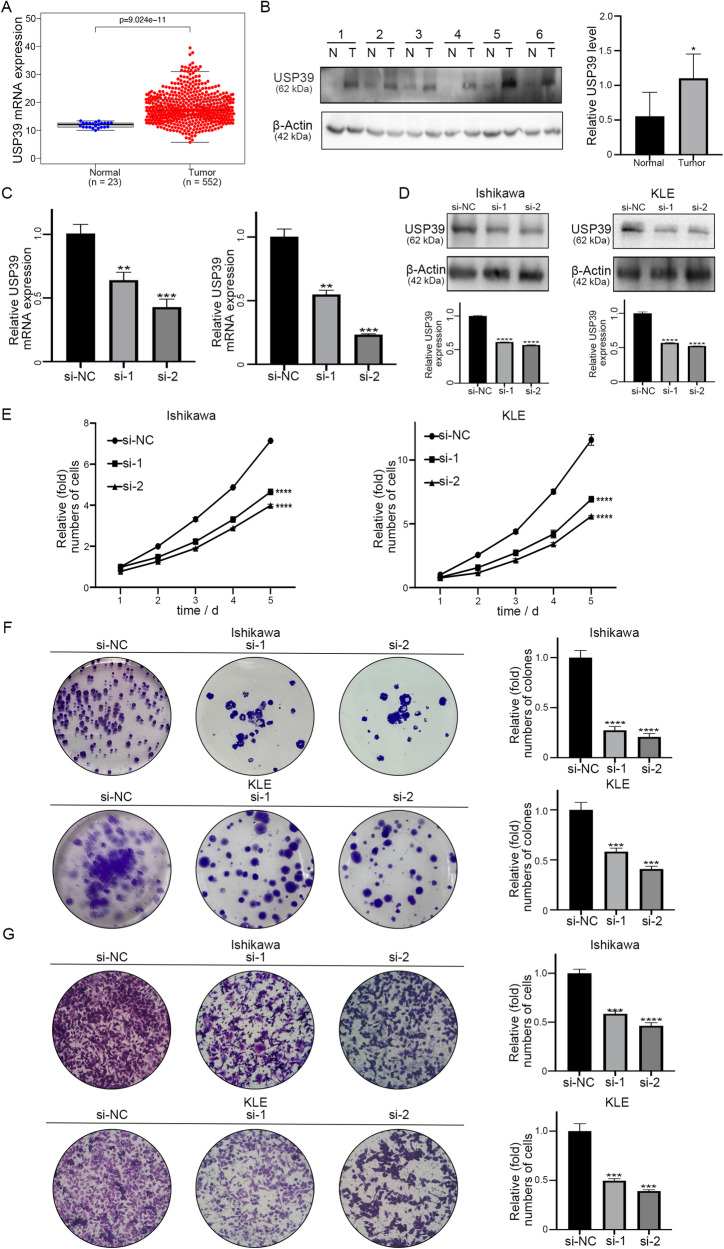
Knockdown of USP39 suppresses cell proliferation and migration of EC cells in vitro. **A** The mRNA expression of USP39 in endometrial tumor tissues and normal endometrial tissues was analyzed based on TCGA databases. **B** USP39 protein expression in EC tissues and adjacent normal tissues was analyzed by western blot. **C** The knockdown efficiency of si-USP39 was detected by qRT-PCR in Ishikawa and KLE cells. **D** The knockdown efficiency of si-USP39 for following experiments was validated by western blot. **E**, **F** Cell proliferation was detected by CCK8 and colony formation assay following USP39 knockdown in EC cells. **G** Cell migration ability was determined by transwell assay following USP39 knockdown in EC cells.

As USP39 can be directly regulated by H3K18la, we next explored USP39 function in EC. First, we performed data mining and analyzed 575 EC cases from The Cancer Genome Atlas (TCGA) database. The expression of USP39 in non-tumor samples (*n* = 23) was much lower than that in EC tumor samples (*n* = 552) (*P* < 0.05; Fig. 4A), and higher expression of USP39 was positively correlated with the clinical grade and FIGO stage of EC (*P* < 0.05; Fig. S5A, B). Moreover, a high USP39 level was associated with a poorer prognosis of patients (*P* < 0.05; Fig. S5C). As Fig. S5D showed, patients with copy-number-high (CN high) had the highest USP39 expression, compared with the risk score of patients with polymerase epsilon (POLE) ultramutated, microsatellite-instable (MSI), or copy-number-low (CN low). The six pairs of tumor specimens and their corresponding adjacent normal tissues from patients with EC were analyzed by western blot. The results indicated that the expression level of USP39 in EC tumor tissue was dramatically higher than that in the normal endometrium (Fig. 4B). Next, EC cell lines were transfected with si-USP39 to downregulate the expression of USP39. The mRNA and protein expression levels of USP39 were significantly decreased in Ishikawa and KLE cells compared with those in the corresponding negative control (Fig. 4C, D). CCK-8 and colony formation assays suggested that the downregulation of USP39 inhibited the proliferation of tumor cells (Fig. 4E, F). In addition, Transwell assays indicated that lower levels of USP39 were associated with a poor migration ability of the cells (Fig. 4G).

**Fig. 5:**
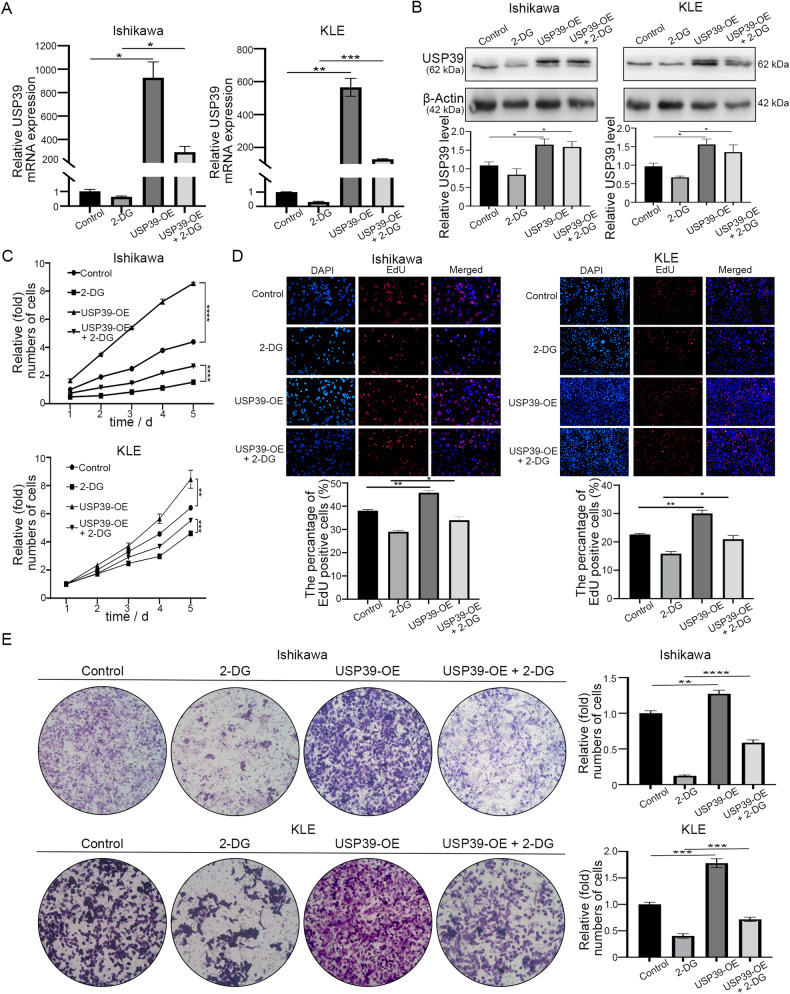
USP39 rescues the reduction of lactylation caused by 2-DG. **A** qRT-PCR showed that USP39 was silenced after 2-DG treatment, upregulated after overexpressing USP39, and could be rescued in EC cells. **B** Western blot showed the USP39 protein level after cocultured with 2-DG and/or USP39 vector. **C** Cell proliferation was assessed by CCK8 assay after down- and/or over-expression of USP39. **D** Images and statistical analysis of cells in the EdU assay were performed using Ishikawa and KLE cells with or without USP39 knockdown. **E** Migration of EC cells with or without USP39 knockdown was analyzed by transwell assay.

We then examined whether the EC tumor suppression effect of the treatment with the histone lactylation inhibitor 2-DG could be reversed by increased USP39 levels. After USP39 was successfully upregulated (Fig. 5A, B), we performed CCK-8 (Fig. 5C) and EdU assays (Fig. 5D), which showed that the growth of EC cells was markedly increased compared with that of control cells. In addition, we observed that the migration ability of EC tumor cells was enhanced after USP39 overexpression, as measured by Transwell assays (Fig. 5E). Moreover, after overexpression of USP39 in EC cells, the 2-DG-induced suppression of USP39 mRNA and protein expression was reversed (Fig. 5A, B), and the anticancer effect of 2-DG was weakened based on the cell growth (Fig. 5C, D) and migration (Fig. 5E). Together, these data suggest that USP39 acts as an oncogene in EC.

### Histone lactylation and USP39 promote EC development, and USP39 overexpression reverses the suppression of tumor growth by 2-DG in vivo

**Fig. 6:**
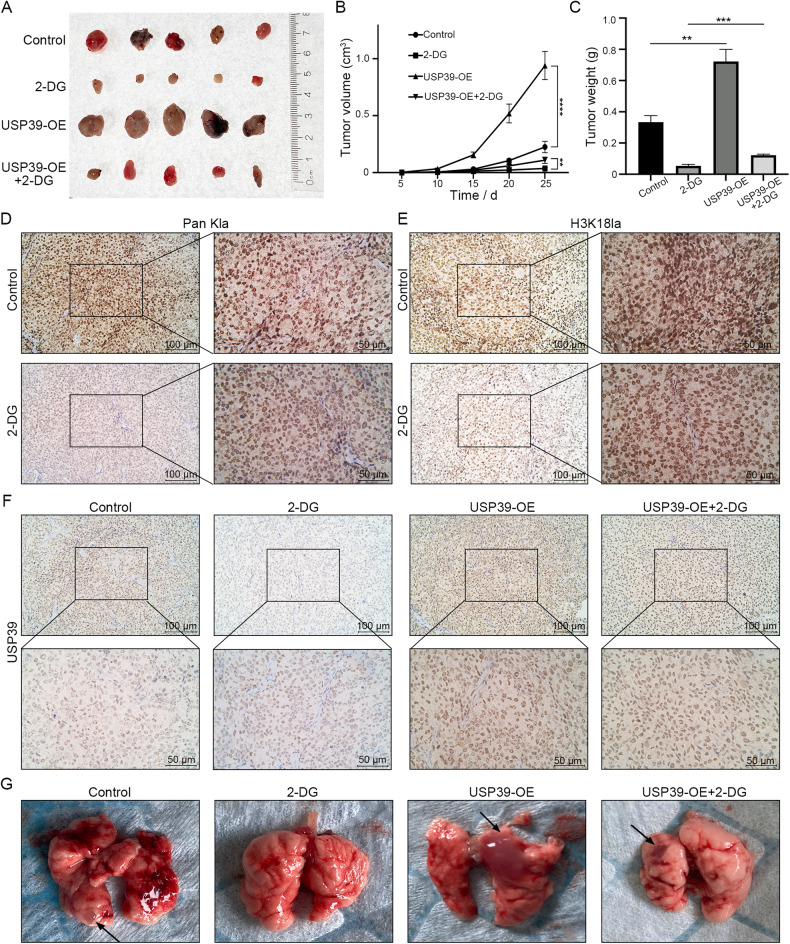
Histone lactylation and USP39 promote EC proliferation and metastasis in vivo. **A** Images of xenograft tumors from BALB/c-nude mice 25 days after the subcutaneous injection of Ishikawa cells. **B** Tumor volume was measured every 5 days and calculated according to the formula: (length × width^2^)/2. **C** Tumor weight of nude mice was assessed on day 25. **D** Immunohistochemistry (IHC) for lactylation modification levels in xenograft tumors. Scale bar: left panel, 100 μm; right panel, 50 μm. **E** IHC for H3K18la levels in xenograft tumors. Scale bar: left panel, 100 μm; right panel, 50 μm. **F** IHC for USP39 expression in xenograft tumors. Scale bar: upper panel, 100 μm; lower panel, 50 μm. **G** Metastatic foci in the lung sections were marked by arrows.

To verify the effect of histone lactylation and USP39 on endometrial cancer, we established an EC xenograft tumor model in nude mice and evaluated the outcomes in vivo. Ishikawa cells pretreated with 2-DG and/or an USP39 vector were subcutaneously injected into nude mice. The tumor size was measured every 5 days until the mice were sacrificed on day 25. As expected, the tumor volume in the group with reduced histone lactylation was significantly inhibited (Fig. 6A, B), and the weight was dramatically lower than that in the control group (Fig. 6C). HE staining showed that Ishikawa cells grew well (Fig. S6A), and IHC showed that the modification levels of Pan Kla and H3K18la significantly decreased after 2-DG treatment (Fig. 6D, E). In addition, overexpression of USP39 accelerated the tumor growth and reversed the 2-DG-caused tumor inhibition (Fig. 6A–C). The IHC results suggested that after 2-DG treatment, the expression level of the USP39 protein in tumors decreased, while the upregulation of USP39 reversed this decrease (Fig. 6F). Subsequently, a tail vein injection-induced EC lung metastasis mouse model was used to evaluate the effect of histone lactylation on EC cell metastasis. As shown in Fig. 6G, no obvious metastatic foci were observed in the lungs after 2-DG treatment, while overexpression of USP39 led to a significantly increased volume of metastatic foci, which could be inhibited by 2-DG treatment. The HE staining results confirmed this trend (Fig. S6B). These data demonstrate that 2-DG can inhibit histone lactylation, thereby weakening EC growth and metastasis, and USP39 overexpression can reverse this tumor-suppressive effect.

### Histone lactylation and USP39 accelerate glycolysis by activating the PI3K/AKT/HIF-1α signaling pathway in EC

**Fig. 7:**
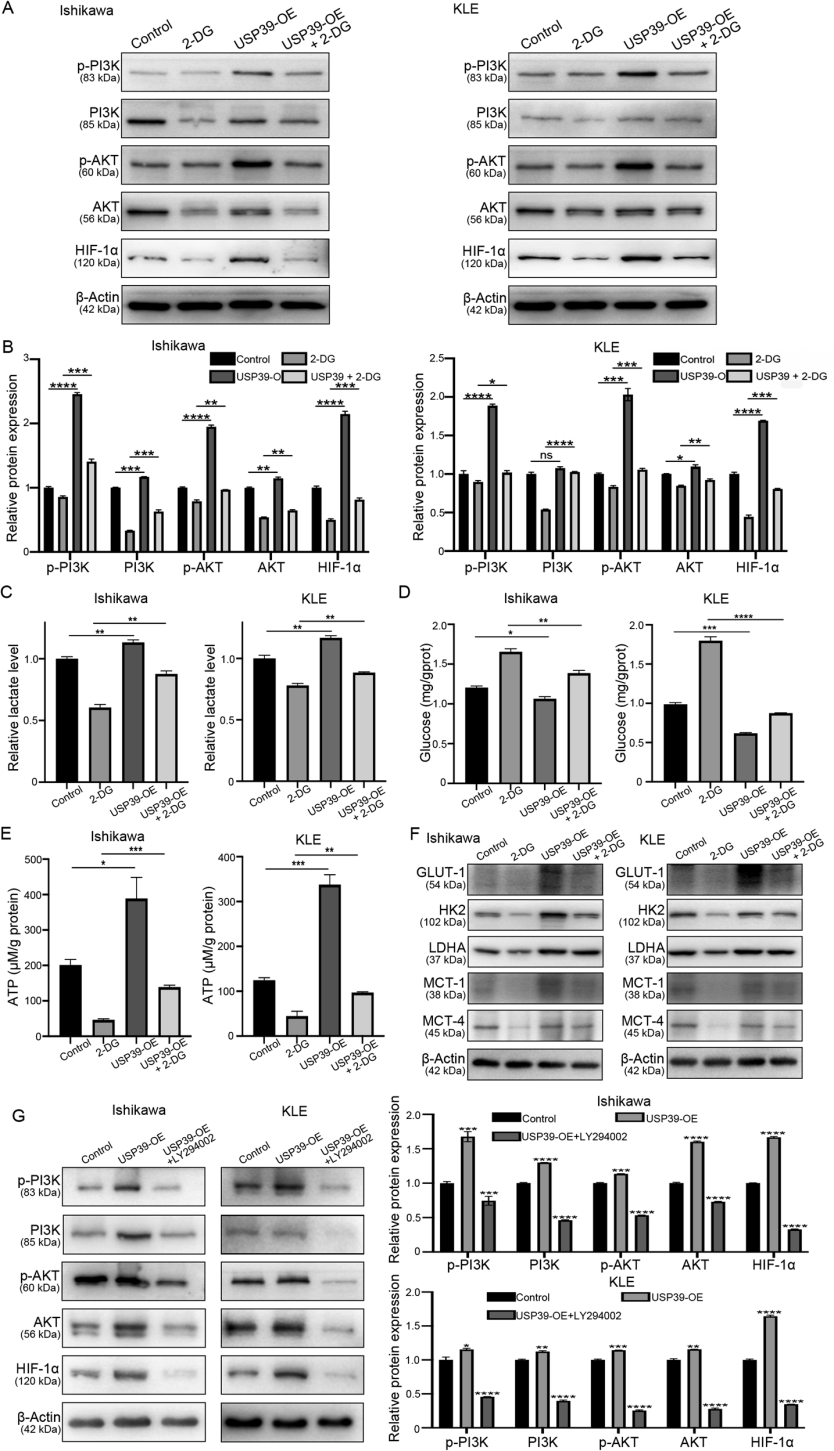
Histone lactylation and USP39 enhance glycolysis via activating PI3K/AKT/HIF-1α signaling pathway. **A** The protein levels of PI3K, p-PI3K, AKT, p-AKT, and HIF-1α in Ishikawa and KLE cells were detected by western blot after being treated with varying conditions. **B** The quantitative analysis of the protein levels of PI3K, p-PI3K, AKT, p-AKT, and HIF-1α. **C** Measurement of intracellular lactate levels in cells treated in varying conditions. **D** Measurement of intracellular glucose in cells treated in varying conditions. **E** Measurement of intracellular ATP levels in cells treated in varying conditions. **F** The expression levels of five glycolysis-related proteins when treated with 2-DG and/or USP39 by western blot. **G** The Protein levels of PI3K, p-PI3K, AKT, p-AKT, and HIF-1α after being treated with LY294002 by western blot.

As KEGG pathway enrichment analysis suggested that the PI3K-AKT and HIF-1 signaling pathways were two of the most significant pathways enriched by USP39, we investigated whether histone lactylation and USP39 could promote the expression of PI3K and its critical downstream protein, HIF-1α, as well as glycolysis, in vitro. The treatment with 2-DG significantly inhibited the PI3K, phospho-PI3K (p-PI3K), AKT, phospho-AKT (p-AKT), and HIF-1α levels in EC cells (Fig. 7A, B). USP39 efficiently induced the phosphorylation of PI3K and AKT and increased HIF-1α expression in EC cells (Fig. 7A, B). In addition, USP39 reversed the 2-DG-induced decreases in the levels of p-PI3K, p-AKT, and HIF-1α (Fig. 7A, B). The lactate levels in Ishikawa and KLE cells were consistent with these trends (Fig. 7C), while the intracellular glucose content showed the opposite trend (Fig. 7D). Moreover, we found that the ATP content decreased with 2-DG treatment and was relatively increased by reversing the USP39 level (Fig. 7E). In addition, we demonstrated by western blotting that the expression of the glycolysis-related proteins GLUT-1, HK2, LDHA, MCT-1, and MCT-4 was decreased by 2-DG treatment and was enhanced by USP39 vectors (Fig. 7F). Next, we used LY294002, a typical PI3K inhibitor, to treat Ishikawa and KLE cells transfected with USP39-expressing vectors. LY294002 effectively reduced the levels of PI3K, p-PI3K, AKT, p-AKT, and HIF-1α, which were elevated by USP39 (Fig. 7G). Collectively, these results indicate that histone lactylation and USP39 promote PI3K/AKT- and HIF-1α-mediated glycolysis in EC cells.

### USP39 stabilizes PGK1 by de-ubiquitination to regulate the PI3K/AKT pathway

**Fig. 8:**
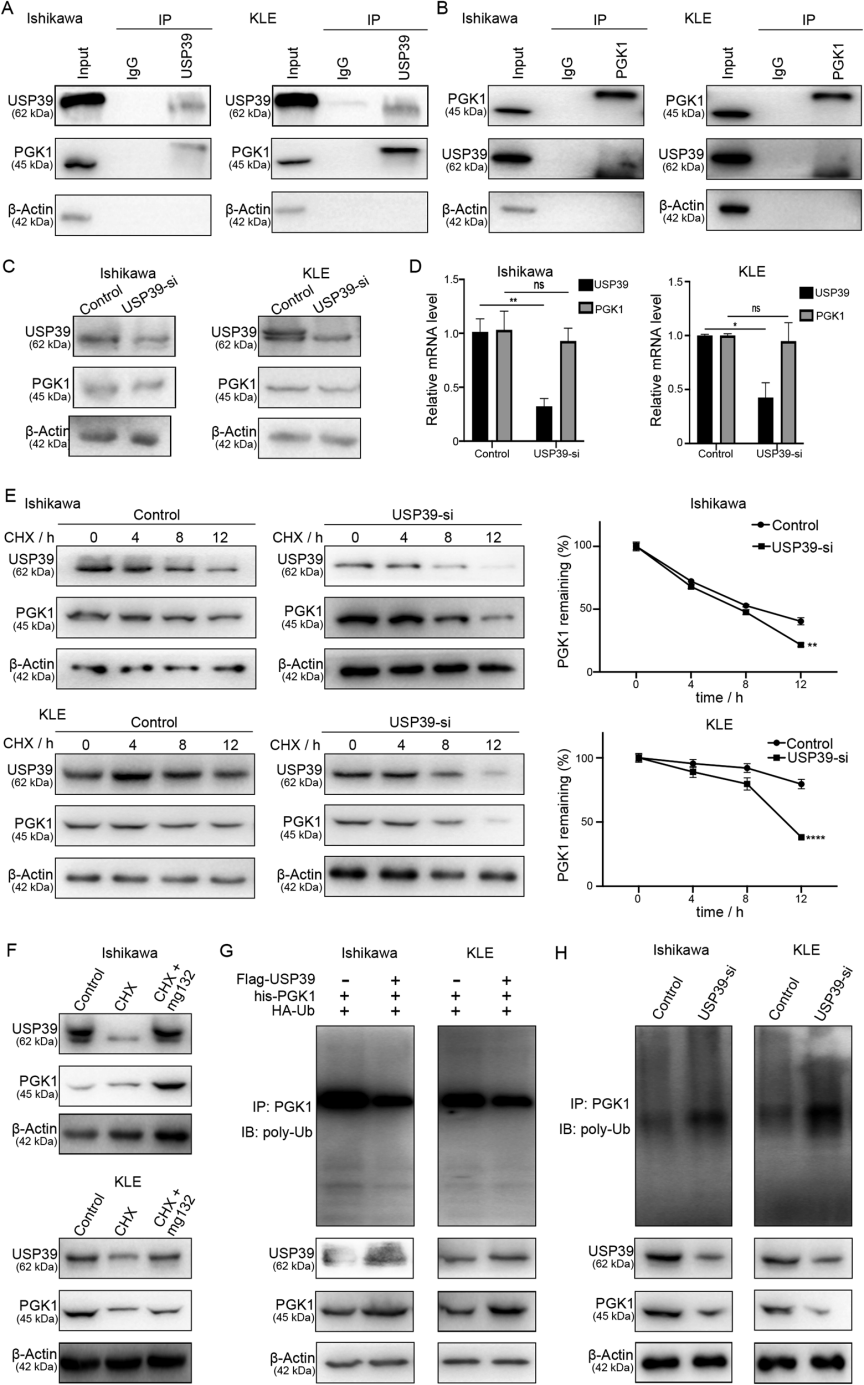
USP39 interacts with PGK1 and stabilizes PGK1 by de-ubiquitination. **A** The immunoprecipitates were blotted with anti-USP39 in Ishikawa and KLE cells. **B** The immunoprecipitates were blotted with anti-PGK1 in Ishikawa and KLE cells. **C** EC cells were prepared for western blotting analysis with anti-USP39 and anti-PGK1 after being transfected with USP39 siRNA. **D** qRT-PCR analysis of the mRNA levels of USP39 and PGK1 in USP39 knocked down cells. **E** The PGK1 level and degradation rate after cycloheximide (CHX) treatment. **F** Western blot was performed with cells treated with mg132. **G** The ubiquitinated forms of PGK1 were analyzed by western blotting analysis with the condition with HA-Ub and his-PGK1 along with Flag-USP39 constructs were co-transfected into Ishikawa and KLE cells. **H** The ubiquitinated forms of PGK1 were analyzed by western blotting analysis after USP39 siRNA was transfected into Ishikawa and KLE cells.

Next, we explored the mechanism of PI3K/AKT pathway regulation by USP39 via analysis of proteins that interacted with USP39. Using Co-IP, followed by mass spectrometry (MS), we identified a number of proteins that could interact with USP39. As shown in Fig. S7A, which presents the MS data, we paid particular attention to candidates that were detected in the USP39 group but not detected in the IgG group. Phosphoglycerate kinase 1 (PGK1), the first ATP-generating enzyme in the glycolytic pathway, has been reported to activate the PI3K/AKT pathway and was chosen for further binding validation by investigating whether endogenous USP39 and PGK1 could interact with each other. A reciprocal IP experiment was conducted in which an endogenous PGK1 protein was co-immunoprecipitated with USP39 (Fig. 8A), indicating that these two proteins could interact endogenously. This result was confirmed by a reciprocal Co-IP assay (Fig. 8B). Moreover, USP39 downregulation resulted in a decrease in the PGK1 protein levels in EC cells (Fig. 8C). To exclude the possibility that the downregulation of the PGK1 protein occurred at the transcriptional level, qRT-PCR was performed to measure the PGK1 and USP39 mRNA levels. In contrast to the significant decrease in the USP39 mRNA level, the PGK1 mRNA level was similar to that in the control cells (Fig. 8D), suggesting that the effect of USP39 on PGK1 was not mediated at the transcriptional level. As USP39 is a de-ubiquitinating enzyme, we explored whether the USP39 de-ubiquitination activity stabilized the PGK1 protein. First, we hypothesized that USP39 extended the half-life of PGK1. PGK1 levels were monitored after treatment with the protein inhibitor cycloheximide (CHX). In the absence of de novo protein synthesis, the half-life of endogenous PGK1 was shorter in USP39-depleted cells than in control cells (Fig. 8E). In addition, the decrease in the PGK1 protein level was reversed by the treatment of Ishikawa and KLE cells with the proteasome inhibitor mg132 (Fig. 8F). HA-ubiquitin and His-PGK1 constructs were co-expressed, with or without Flag-USP39, in Ishikawa and KLE cells. Ectopic expression of Flag-USP39 markedly promoted PGK1 de-ubiquitination (Fig. 8G). Conversely, the USP39 knockdown increased PGK1 ubiquitination in EC cells (Fig. 8H), indicating that USP39 is responsible for PGK1 deubiquitination. Taken together, these data demonstrate that USP39 can stabilize the PGK1 protein by deubiquitination in a proteasome-dependent manner in EC cells.

## Discussion

Epigenetic modifications play a crucial role in the occurrence, development, and metastasis of tumors. The study of histone modifications is expected to further elucidate the mechanisms of tumor progression and metastasis and provide a reliable basis for the research and development of antitumor drugs [27]. Small molecules involved in cell metabolism participate in a variety of epigenetic modifications [28]. In particular, acetyl-CoA [29], *S*-adenosyl methionine [30], and succinate [31] participate as substrates or cofactors in PTM processes such as acetylation, methylation, and succinylation, respectively. Therefore, metabolites play a prevalent and essential role in the epigenetic modification of tumors. As the Warburg effect is one of the hallmarks of cancer, even under aerobic conditions, cancer cells tend to produce lactate via anaerobic glycolysis to generate energy, thus producing more lactate than normal cells at a given time [32]. Used as a metabolic byproduct, lactate has been discovered to alter histones by the addition of the lactyl group, which is described as histone lactylation. Histone lactylation is caused by lactate, and the process is sensitive to lactate levels. Studies have shown that under hypoxia or bacterial infection, intracellular glucose is not completely oxidized, and the produced metabolite lactate stimulates the histone lactylation modification and activates downstream gene expression [33–35]. With regard to the broader roles of this modification, lactate is generated by cells under both physiological conditions and disease states, such as cancer. Histone lactylation participates in cellular communication and regulates the immune system [36], links cellular metabolism to gene regulation, and may have numerous implications for human health. Owing to the active glycolysis and lactate metabolism processes in EC, we suspect that histone lactylation in EC is likely to be abnormal. Thus, exploration of the potential role of histone lactylation in the occurrence and development of EC tumors is of great significance.

MS analysis identified 26 histone Kla sites on core histones, including H3K9, H3K18, and H3K28, from human MCF-7 cells [19]. Most studies have focused on H3K18, which may be due to the imperfect development of corresponding antibodies to other sites. Gene Ontology analysis has shown that H3K18la-specific genes were enriched in biological pathways unrelated to inflammation, including the induction of arginase 1 expression [19].

In this study, we first demonstrated by IHC and western blotting that global lactylation was increased in EC. In vitro experiments showed that glycolysis inhibitors, 2-DG and oxamate, reduced the global lactylation levels and those at a selected Kla site, H3K18la, in a concentration-dependent manner. It was also found that after treatment with 2-DG and oxamate, the proliferation and migration of EC cells were significantly inhibited, while the rate of apoptosis was dramatically increased, and the cell cycle was effectively blocked. In addition, in vivo studies using mice models showed that decreasing lactylation significantly reduced the volume and weight of tumors and suppressed metastasis. These data confirm the fundamental role of histone lactylation as a new target for the prevention of EC tumor development.

To explore the potential downstream targets of histone lactylation, we performed RNA-seq to compare the mRNA levels between control and EC cells treated with 2-DG. Consequently, we selected genes that were highly expressed in EC tumor cells and whose mRNA levels were significantly reduced in 2-DG-treated cells. Among multiple candidate genes, we focused on USP39, which has been reported to act as a carcinogen in various tumors. Deubiquitinases are a large protease superfamily [37], among which the ubiquitin-specific protease family (also known as ubiquitin-specific processing enzymes) has the largest number of members and the most diverse structure [38]. This family contains two short conserved fragments, lysine and histidine boxes. The sequence contains a catalytic triad of cysteine, histidine, and aspartic acid or asparagine residues, which can remove ubiquitin molecules from large proteins [39]. Thus, USP15 is related to the COP9 signalosome complex and is involved in the regulation of a variety of signaling pathways [40]. In the base excision repair pathway, USP47 deubiquitinates and stabilizes monoubiquitinated DNA polymerase β [41]. USP30 can remove ubiquitin tags and participate in the regulation of mitochondrial morphology in cells, thus inhibiting mitochondrial autophagy [42]. Previous studies have shown that USP39 is significantly involved in the occurrence and development of cancer. In particular, USP39 de-ubiquitinates and stabilizes CHK2 to regulate the DNA damage response and chemical radiation resistance [43], de-ubiquitinates and stabilizes the SP1 protein to promote hepatocellular carcinoma progression [44], and inhibits VEGF-A165b-selective splicing by regulating SRSF1 and SRPK1 to promote malignant proliferation and angiogenesis of renal cell carcinoma [45].

In this study, we further explored whether H3K18la could regulate the expression of USP39. We then performed ChIP-qPCR and EMSA analysis, and the results showed that H3K18la was enriched in the USP39 promoter region, and this enrichment was efficiently reduced by 2-DG. Next, we confirmed that USP39 was upregulated in EC by analyzing online databases and clinical samples. Using CCK8, EdU, and colony formation assays, as well as in vivo trials, USP39 was shown to promote EC proliferation and migration. As the PI3K/AKT pathway is common in various cancers [46], we verified whether USP39 could stimulate PI3K/AKT/HIF-1α signaling to accelerate glycolysis.

As USP39 is a deubiquitinase, we investigated its interacting proteins. Based on the MS results, followed by Co-IP, we focused on PGK1, the first ATP-generating enzyme in the glycolytic pathway and a HIF-1-targeted gene [47–49], which has been reported to activate the PI3K/AKT pathway [50]. A reciprocal IP experiment and a reciprocal assay confirmed that PGK1 and USP39 interacted with each other. In addition, USP39 deubiquitinated and stabilized PGK1. Most importantly, USP39 regulated the PI3K/AKT/HIF-1α signaling pathway via interaction with PGK1.

Our study has some limitations. Only twelve pairs of EC and normal tissue samples were available, and the only pathological type was endometrioid adenocarcinoma. Expanding the number of tissue samples and pathological types of EC and following up on prognosis should be considered. Finding compounds that can directly target lactylation sites could help elucidate the mechanism of histone lactylation in EC. Further experiments were required to establish the role of H3K18la in the transcriptional activation of the corresponding gene USP39.

## Conclusion

In conclusion, we explored histone lactylation in EC (Fig. S8) and found that lactate production in EC stimulated histone lactylation. In turn, histone lactylation regulated the expression of USP39, and USP39 activated the PI3K/AKT/HIF-1α signaling pathway by interacting with PGK1. Finally, glycolysis was stimulated to produce more lactate, which further increased histone lactylation. Thus, histone lactylation may be a promising direction for research and a potential therapeutic target for EC.

### Supplementary information

Additional file 1: Fig. S1. The modification levels of five lactylation sites in EC tissues and adjacent normal tissues. Fig. S2. IC50 of 2-DG and oxamate in Ishikawa and KLE cells. Fig. S3. The 2-DG and oxamate treatment suppresses migration, induces apoptosis, and arrests cell cycle progression of EC cells in vitro. Fig. S4. GO and KEGG enrichment analysis of differentially expressed genes from RNA-seq. Fig. S5. The analysis of TCGA database about USP39. Fig. S6. Images of HE staining for xenograft tumors and metastatic foci in the lung sections. Fig. S7. The image of sliver staining to identify the interacting proteins. Fig. S8. Mechanism diagram.

## Methods

### Cell culture

Five types of human EC cell lines (Ishikawa, RL95-2, HEC-1A, HEC-1B, and KLE) were obtained from the American Type Culture Collection (ATCC, USA). The normal endometrial cells we used were human primary endometrial epithelial cells. Cells were grown in DMEM/F12 complete medium (HyClone, China) supplemented with 10% fetal bovine serum (FBS; Gibco, USA) and penicillin and streptomycin (Boster, China) at final concentrations of 100 µg/mL. All cells were free from *Mycoplasma* contamination and were grown at 37 °C in a humidified atmosphere of 5% CO_2_.

### Clinical specimens

A total of 12 paired EC and adjacent normal tissues of patients who underwent surgery or biopsy at the Department of Gynecology, Union Hospital Affiliated to Tongji Medical College, Huazhong University of Science and Technology (Wuhan, China) from September 2019 to March 2021 were collected for immunohistochemistry (IHC) and western blot. All patients had complete clinical data and did not receive immunotherapy, chemotherapy, or radiotherapy. This study was approved by the Ethics Committee of Tongji Medical College, Huazhong University of Science and Technology (No. 2022-S017).

### Bioinformatical analysis

TCGA (http://www.cancergenome.nih.gov/) was used for gene set enrichment analysis (GSEA) to analyze the expression and prognosis of USP39 in patients with EC. The ggplot2 package in R was applied to perform a volcano plot of all the differentially expressed genes based on the RNA-seq results.

### Immunohistochemistry (IHC) staining and scoring

Samples were embedded in paraffin and sliced into sections at a thickness of 4 μm. The sections were then incubated with anti-L-lactyl lysine rabbit mAb (pan-anti-Kla; PTM-1401, PTM Bio, China), anti-lactyl-histone H3 (Lys18) rabbit mAb (anti-H3K18la; PTM-1406, PTM Bio, China), and anti-USP39 (23865-1-AP, Proteintech, China) at 4 °C overnight. The slides were then rinsed three times with PBS, incubated with a secondary antibody at room temperature for 30 min in the darkness, and visualized after being stained with a DAB solution. Three randomly selected fields were observed under a microscope (Motic, China). The IHC staining scores, based on the staining intensity (SI) and the percentage of immunoreactive cells (PR), were evaluated by two independent observers who were blinded to the patient’s identity. The SI scores were assigned from 0 to 3 as follows: 0 = no staining; 1 = weak staining; 2 = moderate staining; and 3 = strong staining. The PR was scored from 1 to 4 as follows: 1 = 0–25%; 2 = 26–50%; 3 = 51–75%; and 4 = 76–100%. The PR and SI scores were multiplied to produce a weighted score for each patient. A score of 8–12 was defined as a high expression level, and a score of 0–7 was defined as a low expression.

### Total RNA isolation and quantitative real-time polymerase chain reaction (qRT-PCR)

Adherent cells were harvested with the RNAiso reagent (Takara, Japan), and RNA was extracted according to the manufacturer’s manual. The concentration of RNA was measured using a Tecan Infinite M200 Pro (Thermo Fisher Scientific, USA). cDNA was obtained from the RNA using a reverse transcription kit (Vazyme, China), and was used as a template for qRT-PCR with the SYBR Green Fast qPCR mix (ABclonal, China). The fold changes of RNA transcripts were calculated using the 2^−ΔΔCt^ method, and β-Actin was used as an endogenous control. All qRT-PCR runs were conducted in triplicate. The primer sequences used were as follows: USP39-F: 5′-GGTTTGAAGTCTCACGCCTAC-3′; USP39-R: 5′-GGCAGTAAAACTTGAGGGTGT-3′; β-Actin-F: 5′-GGATTCCTATGTGGGCGACG-3′; and β-Actin-R: 5′-GATAGCACAGCCTGGATAGCA-3′.

### Western blot

Fresh tissues or adherent cells were washed twice with PBS and then lysed on ice for 1 h with RIPA lysis buffer (Servicebio, China) containing a cocktail and PMSF. After centrifugation, total protein concentrations were determined using a BCA protein assay kit (Biosharp, China). Individual samples (20 μg/lane) were separated by sodium dodecyl sulfate (SDS)-polyacrylamide gel electrophoresis on 10–12% gels and transferred onto polyvinylidene difluoride membranes. After incubation with 5% dry milk in TBST at room temperature for 1 h, the membranes were washed and incubated with pan-anti-Kla (PTM-1401, PTM Bio, China), anti-H3K18la (PTM-1406, PTM Bio, China), anti-USP39 (23865-1-AP, Proteintech, China), anti-PGK1 (17811-1-AP, Proteintech, China), anti-PI3K (T40115, Abmart, China), anti-p-PI3K (T40116, Abmart, China), anti-AKT (T55561, Abmart, China), anti-p-AKT (T40067, Abmart, China), anti-HIF-1α (TA1009, Abmart, China), anti-β-Actin (AC038, ABclonal, China), and anti-H3 (17168-1-AP, Proteintech, China) at 4 °C overnight. After washing the membranes, the bound antibodies were detected with horseradish peroxidase-conjugated secondary antibodies and visualized using an enhanced chemiluminescence (ECL) kit (Biosharp, China). The relative expression or modification levels of the targets to those of β-Actin or H3 were determined by densitometric analysis using the ImageJ software.

### Glucose uptake, lactate accumulation, pyruvate content, and ATP content assays

EC cells were seeded into six-well plates and treated with 2-deoxy-D-glucose (2-DG), oxamate, or a plasmid for 24 h. Afterward, the cells were washed twice with PBS and lysed with a lysis buffer. The concentrations of glucose, lactate, pyruvate, and ATP were determined using commercial assay kits (S0201S, Beyotime, China; A019-2-1, NJJCBIO, China; A081-1-1, NJJCBIO, China; A095-1-1, NJJCBIO, China), according to the manufacturer’s instructions.

### Half maximum inhibitory concentration (IC50) determination

3 × 10^3^ cells were seeded into 96-well plates and placed in the incubator for cell adhesion. According to the experimental settings, the drugs were adjusted to different concentrations and added 200 μL to each well. After treatment for 24 h, 100 μL serum-free medium containing 10% CCK-8 solution was added to each well and continued to incubate for 1 h. The absorbance of each well was measured at 450 nm and relative cell inhibition rates were calculated. After converting the drug concentration to log10, the fitting curve was drawn to obtain the drug action concentration when inducing 50% cell death.

### Cell counting kit-8 assay

Cell Counting Kit-8 (CCK-8; C0005, Targetmol, China) was used to measure cell proliferation. Cells were seeded into 96-well plates at a density of 3 × 10^3^ cells per well in a 100-µL suspension. Every 24 h for 5 days, 10 μL of the CCK-8 solution was added to each well in 100 μL of a serum-free medium. After incubation for 2 h at 37 °C, the absorbance of each well was measured at 450 nm using a microplate reader (Thermo Fisher Scientific, USA). Finally, the numbers of living cells over 5 days were plotted in a graph to reflect the cell proliferation rate.

### Colony formation assay

For the colony formation assay, cells were seeded into six-well plates at a density of 1 × 10^3^ cells per well and cultured for 14 days. Subsequently, the cells were washed with PBS, fixed with 1 mL of methanol for 30 min, and stained with a crystal violet solution for approximately 30 min. After being washed and air dried, the colonies formed were counted manually.

### 5-Ethynyl-2′-deoxyuridine (EdU) assay

The EdU assay was performed with Ishikawa and KLE cells using an EdU-555 cell proliferation kit according to the manufacturer’s protocol (C0075S, Beyotime, China). Cells were seeded into six-well plates, and 24 h later, a 10 μM EdU solution was added to the cells for 2 h. Thereafter, the cells were fixed with 4% paraformaldehyde and rinsed three times with PBS and once with 0.5% Triton X-100. Next, a click additive reaction system was added to label the proliferated cells, and Hoechst 33342 was added for cell counting. Finally, cells were visualized using a fluorescence microscope (Olympus, Japan).

### Cell migration assay

Transwell 24-well plates with an 8-μm pore size (Corning, USA) were used to assess cell migration. Endometrial cells were treated with histone lactylation inhibitors for 24 h firstly. Serum-free culture medium (100 µL) was added to the upper chamber with Ishikawa or KLE cells (1.0 × 10^5^/well), and 700 μL of a medium with 20% FBS as a chemo-attractant was placed into the lower compartment of the chamber. After 24 h incubation at 37 °C, cells that crossed the membrane were fixed with 4% paraformaldehyde (Servicebio, China) for 30 min and stained with 0.1% crystal violet (Servicebio, China) for 10 min at room temperature. Migrated cells were imaged and counted in three randomly selected fields of view using a CX23 light microscope (Olympus, Japan) with a 100× magnification.

### Flow cytometry (FCM) analysis

An annexin V-FITC apoptosis detection kit (C1062M, Beyotime, China) was used to evaluate cell apoptosis, and a PI/RNase staining buffer (550825, BD Biosciences, USA) was used to analyze the cell cycle. The flow cytometer used was an ID7000 spectral cell analyzer (Sony, Japan), and the data were analyzed using the FlowJo software.

### Chromatin immunoprecipitation (ChIP)-qPCR

Ishikawa and KLE cells (3 × 10^6^) were seeded into 100-mm dishes, and after treatment with 1% formaldehyde, the cells were lysed with 1 ml of RIPA lysis buffer. Genomic DNA was isolated and sheared into 200–400-bp fragments using a sonicator. After centrifugation, the supernatants were collected, and the chromatin was precipitated with anti-H3K18la (1:50; PTM-1406, PTM Bio, China) or IgG (30000-0-AP, Proteintech, China) at 4 °C overnight. The following steps were conducted according to the manufacturer’s instructions for a ChIP assay kit (P2078, Beyotime, China). The primers designed for specific promoter regions of *USP39* are listed in Supplementary Table 1.

### Electrophoretic mobility shift assay (EMSA)

Nuclear extracts were prepared from Ishikawa and KLE cells using a Nuclear and Cytoplasmic Protein Extraction Kit (P0027, Beyotime, China). The gel shift binding reaction was performed according to the instructions provided with the Chemiluminescent EMSA Kit (GS009, Beyotime, China). Samples were run on non-denaturing polyacrylamide gels and transferred to a Nylon membrane. After cross-linking, the biotin-labeled DNA was detected and visualized. Reduction in the mobility of DNA-protein complexes on the gels represented the binding of histone to the USP39 promoter. The primer sequences of biotin-labeled probes were as follows: USP39 c site: Bio-5′-AGCTGCGGCCCTCGGAGCAGCCCTGAAAGGTTTAAAGGGCCGA-3′ and USP39 d site: Bio-5′-AAGTTGAGGTCCCTAAGGCTTGATGCCACACCAGCACCTGCAG-3′.

### Co-immunoprecipitation (Co-IP) assay and mass spectrometry (MS)

For immunoprecipitation (IP), cells were harvested and lysed with NP40 buffer (Servicebio, China), followed by the addition of PMSF and a protease inhibitor cocktail. After centrifugation, the supernatant was collected in fresh tubes. Approximately 1 mg of the cell lysate was incubated with 1.0 μg of primary antibodies (anti-IgG and anti-USP39) with a rotation overnight at 4 °C. Then, 25 μL of washed protein A agarose beads (HY-K0202, MCE, USA) were added to the lysate, and the incubation continued for 2 h at 4 °C with a rotation. Subsequently, the beads were collected by centrifugation and washed three times with NP40 buffer. The immune complex was released by boiling the beads with an SDS loading dye and analyzed by western blot. Purified peptides were pickup by autosampler and transferred into a C18 analytical column (Thermo Fisher Scientific, USA) for separation. A Q Exactive Plus mass spectrometer coupled with an EASY-nLC 1200 system (Thermo Fisher Scientific, USA) was used to acquire LC-MS/MS data and the MS raw data were analyzed with MaxQuant (V1.6.6) using the Andromeda database search algorithm, filtered with 1% FDR at both protein and peptide levels.

### In vivo tumor formation

After random grouping into an experimental and control group, SPF BALB/c-nu female nude mice were subcutaneously injected with treated Ishikawa and normal Ishikawa cells (1 × 10^6^), respectively. The tumor size was measured every 5 days, and then the mice were killed by cervical dislocation on day 25. The xenograft volume was calculated using the following formula: tumor volume (mm^3^) = (length × width^2^)/2. The tumors were weighed and embedded in paraffin for an IHC assay. Animal experiments were performed according to the protocols approved by the Animal Care and Use Committee of the Tongji Medical College (2021-S2783). For animal studies, the operation should obey blinding.

### Mouse model of EC lung metastasis

After random grouping, SPF BALB/c-nu female nude mice were injected via the tail vein with Ishikawa cells (1 × 10^6^ in 0.1 ml of PBS). After 6 weeks of treatment, the mice were killed by cervical dislocation. The lungs were excised, fixed, and embedded in paraffin to observe probable lung metastasis. For animal studies, the operation should obey blinding.

### Statistical analysis

GraphPad Prism 8 (GraphPad Software, USA) was used for statistical analysis. Experimental data from three independent replicates are presented as the mean ± standard deviation (SD). Student’s *t* test was used for comparisons between two independent sample groups with a normal distribution of data in qRT-PCR, Western blot, IHC scoring, colony formation assay, EdU assay, Transwell assay, glucose uptake assays, lactate accumulation assays, pyruvate content assays, ATP content assay, and flow cytometry analysis, et al. A one-way analysis of variance was used for group comparisons of all points in the curves when conducting CCK-8 assay and xenograft volume analysis. Overall survival curves were plotted via the Kaplan–Meier method and compared by the log-rank test. *P* < 0.05 was considered to indicate a statistically significant difference. *P < 0.05, **P < 0.01, ***P < 0.001, ****P < 0.0001.

### Supplementary information

Additional file 1

Additional file 2 Table S1. Primers designed for specific promoter regions of USP39.

Additional file 3 Table S2. Differentially expressed genes of RNA-Seq.

Additional file 4 Uncropped western blotting analysis.

## Data Availability

The raw data and materials supporting the conclusions of this article will be made available by the authors, without undue reservation.
