# Supplementary material for: Histone lactylation promotes malignant progression by facilitating USP39 expression to target PI3K/AKT/HIF-1α signal pathway in endometrial carcinoma
Source: Cell Death Discov. 2024 Mar 8;10:121. doi: 10.1038/s41420-024-01898-4 (PMC10923933; doi:10.1038/s41420-024-01898-4)
Supplement: Supplementary file 1 — Additional file 1 [file 41420_2024_1898_MOESM1_ESM.pdf]

Supplement Figure 1.

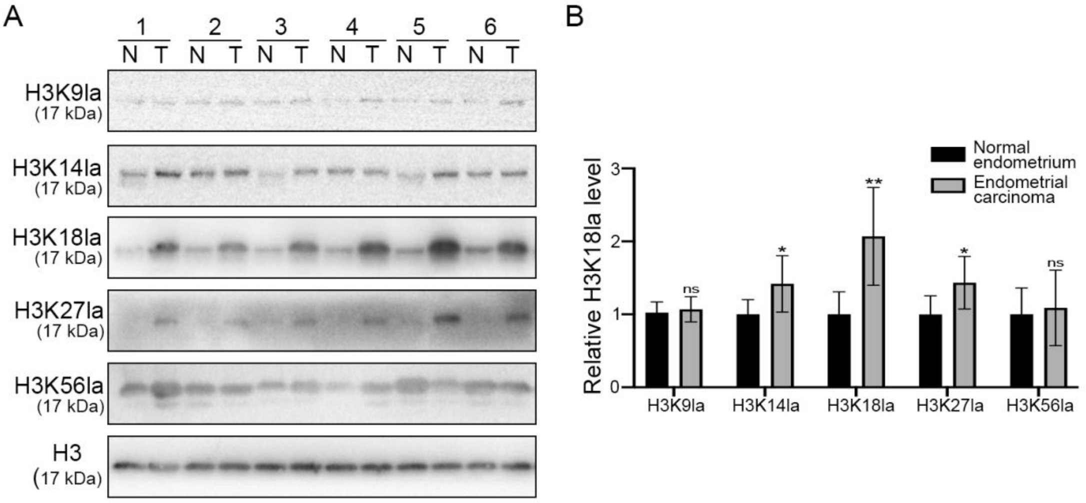

The modification levels of five lactylation sites in EC tissues and adjacent normal tissues. (A) Five histione lactylation sites modification levels in EC tissues and adjacent normal tissues were analyzed by western blot. (B) The levels of lactylation sites in EC tissues and adjacent normal tissues were visualized.

Supplement Figure 2.

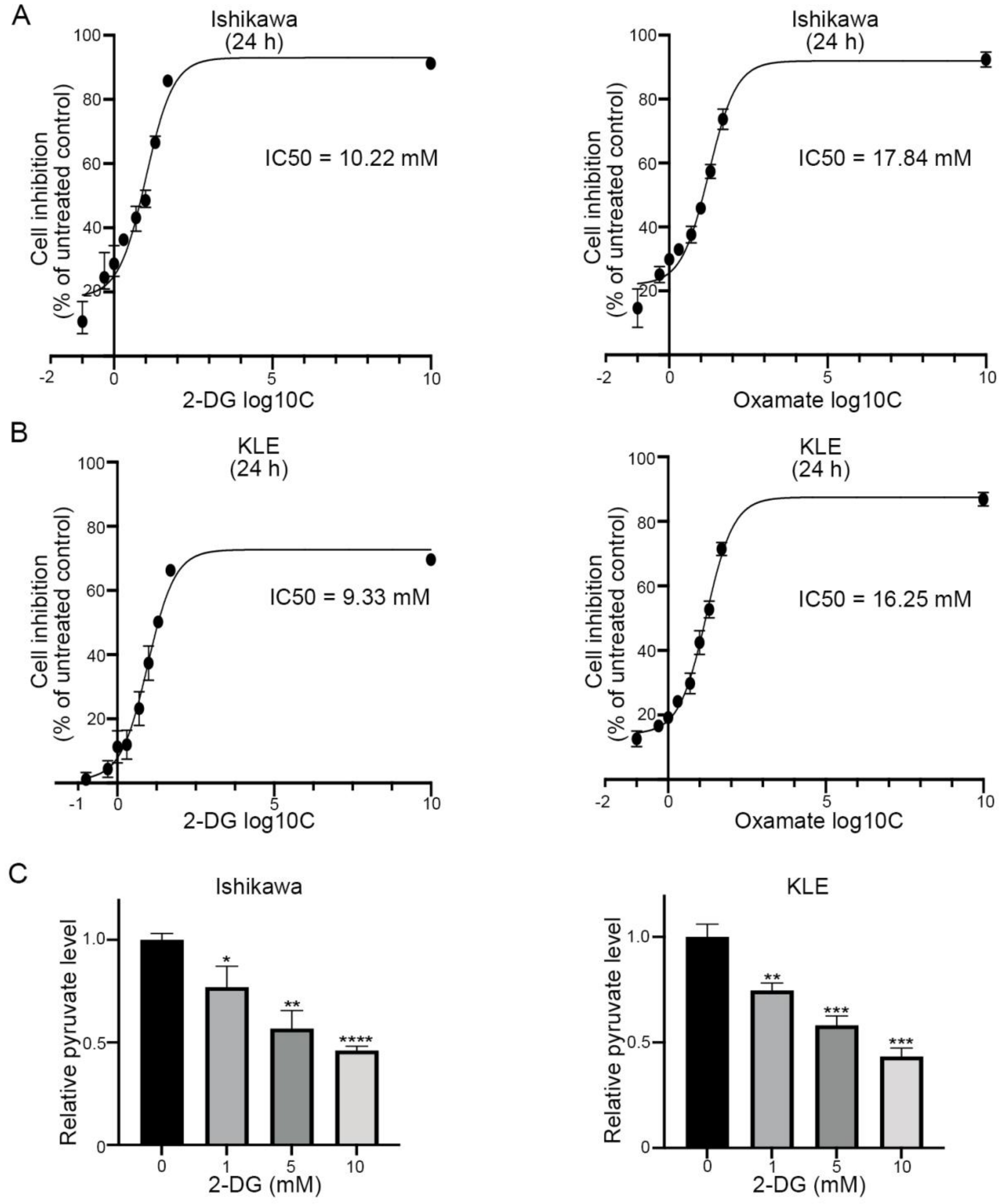

Half maximal inhibitory concentration (IC50) of 2-DG and oxamate in Ishikawa and KLE cells. (A) The IC50 of 2-DG and oxamate in Ishikawa cells. (B) The IC50 of 2-DG and oxamate in KLE cells. (C) Intracellular pyruvate levels were measured from Ishikawa and KLE cells cultured in different concentrations of 2-DG.

Supplement Figure 3.

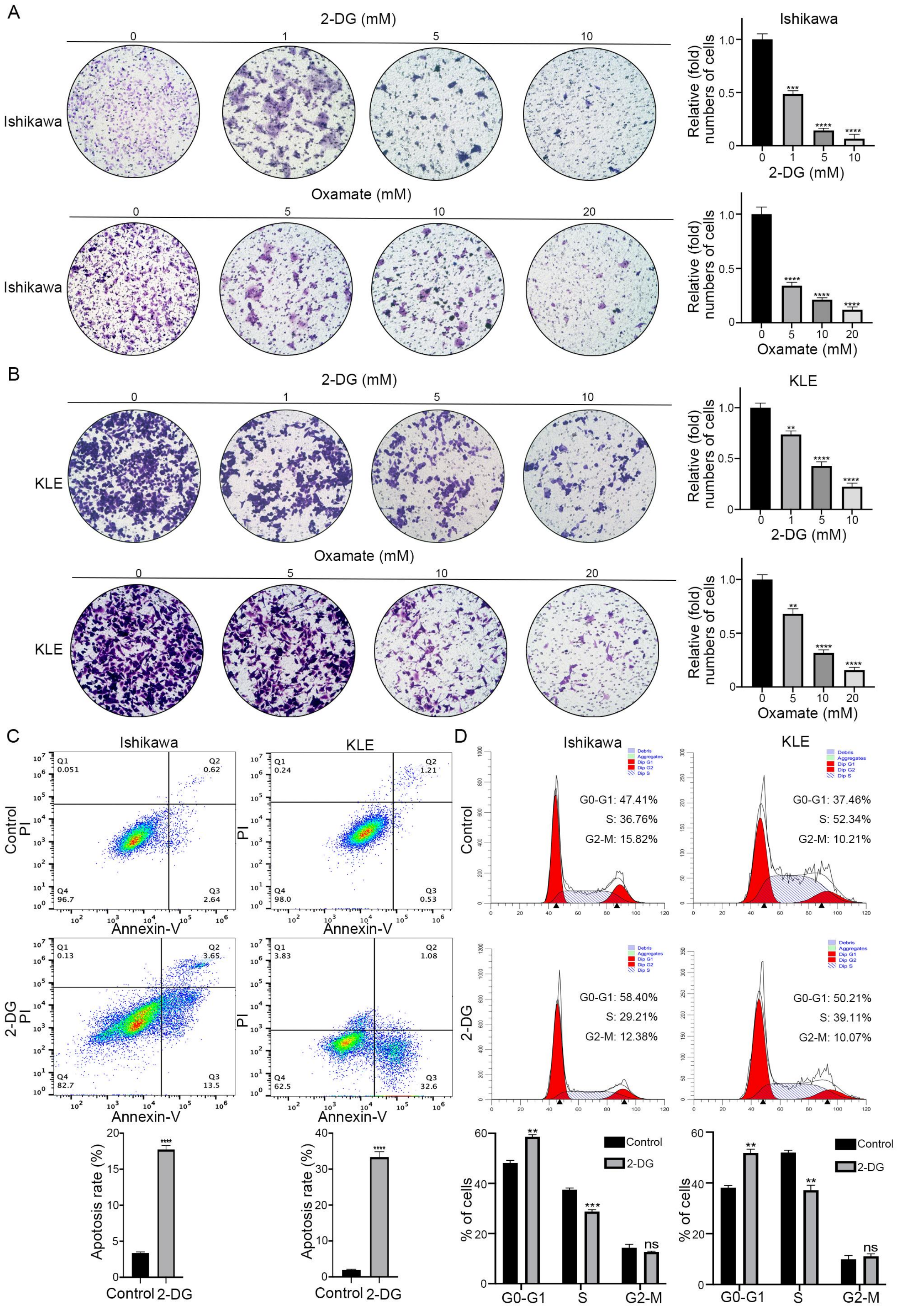

The 2-DG and oxamate treatment suppresses migration, induces apoptosis, and arrests cell cycle progression of EC cells in vitro. (A, B) Cell migration ability was determined by transwell assay following 2-DG or oxamate treatment in EC cells. (C)

Cell apoptosis of EC cells following 2-DG treatment was analyzed by a flow cytometer. (D) The cell cycles of Ishikawa and KLE cells treated with 2-DG were analyzed using flow cytometry.

Supplement Figure 4.

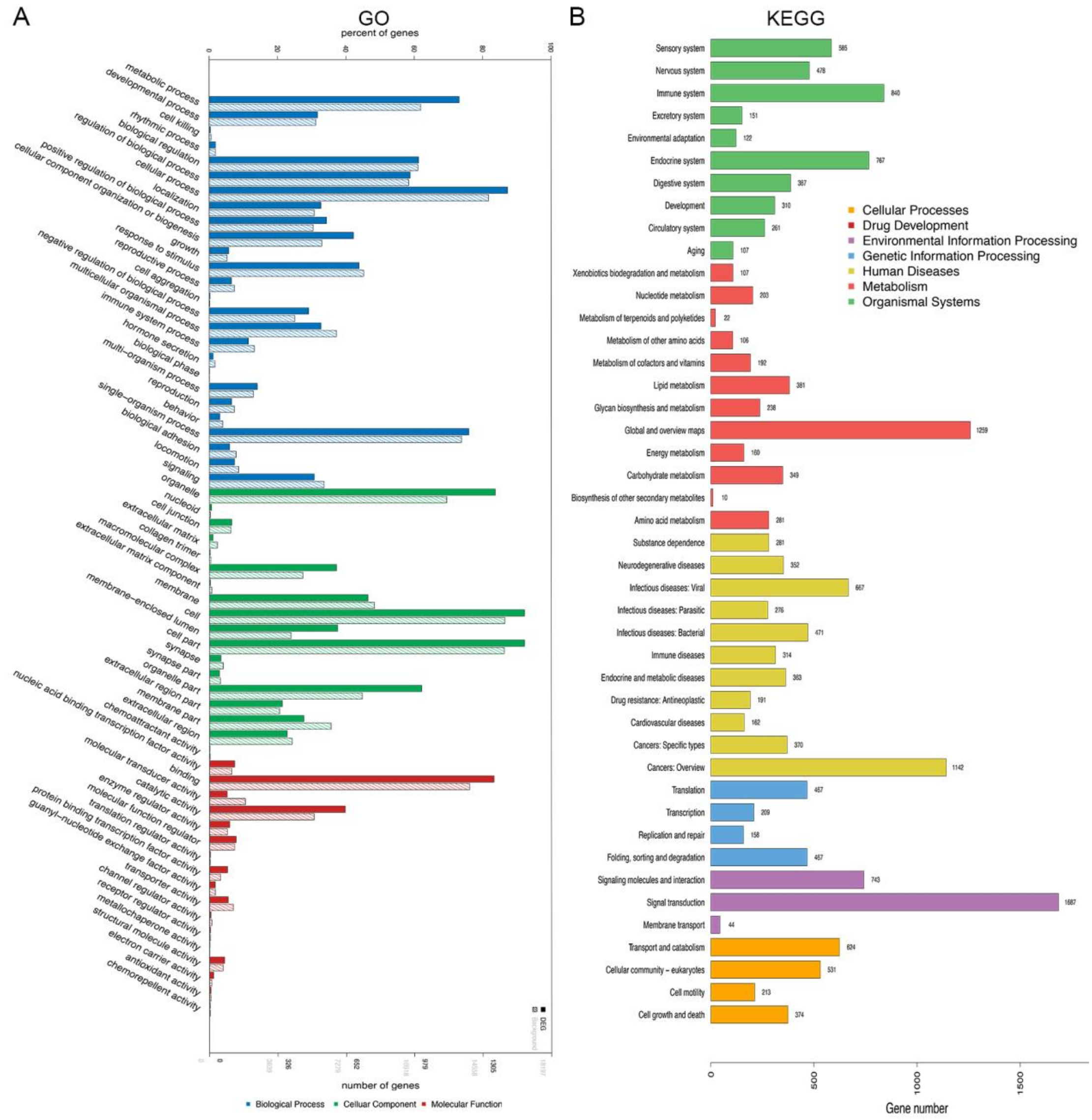

GO and KEGG enrichment analysis of differentially expressed genes from RNA-seq. (A) GO enrichment analysis of differentially expressed genes from RNA-seq. (B) KEGG enrichment analysis of differentially expressed genes from RNA-seq.

Supplement Figure 5.

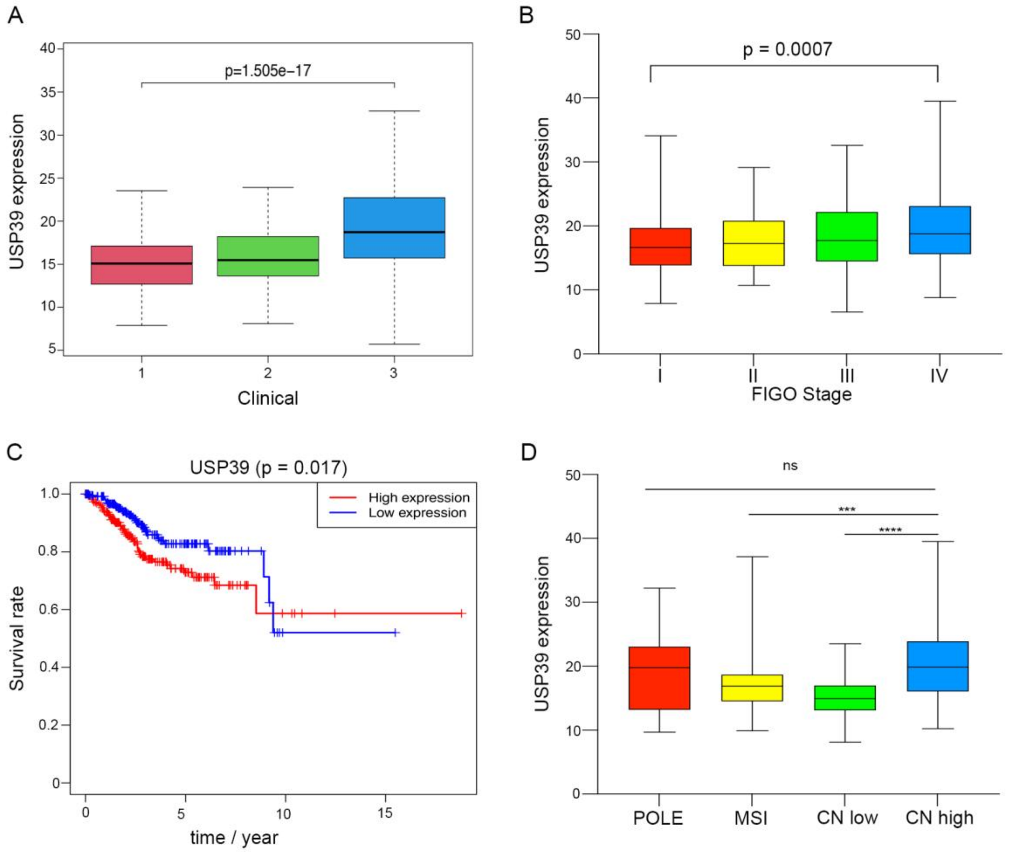

The analysis of TCGA database about USP39. (A) The expression of USP39 was positively correlated with EC clinical-grade (P<0.05). (B) USP39 expression was positively correlated with EC FIGO stage (P<0.05). (C) Higher USP39 levels were associated with a poorer prognosis (P < 0.05). (D) USP39 expression among four different subtypes of EC.

Supplement Figure 6.

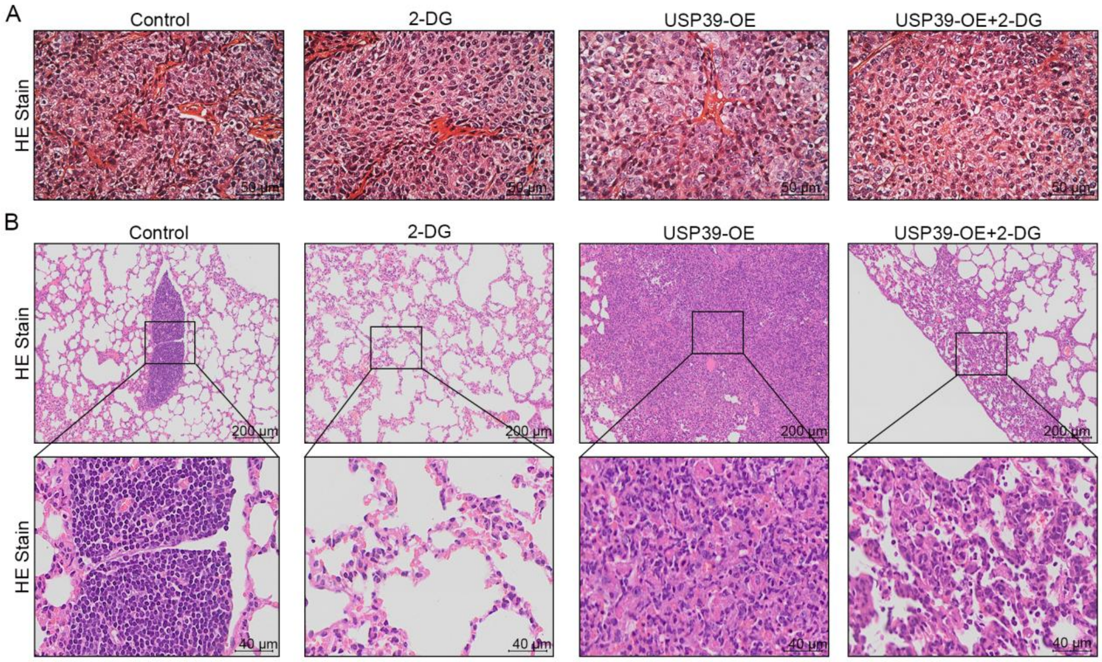

Images of HE staining for xenograft tumors and metastatic foci in the lung sections. (A) Images of HE staining for xenograft tumors. Scale bar: 50 μm. (B) Images of HE staining for metastatic foci in the lung sections. Scale bar: upper panel, 200 μm; lower panel, 40 μm.

Supplement Figure 7.

A

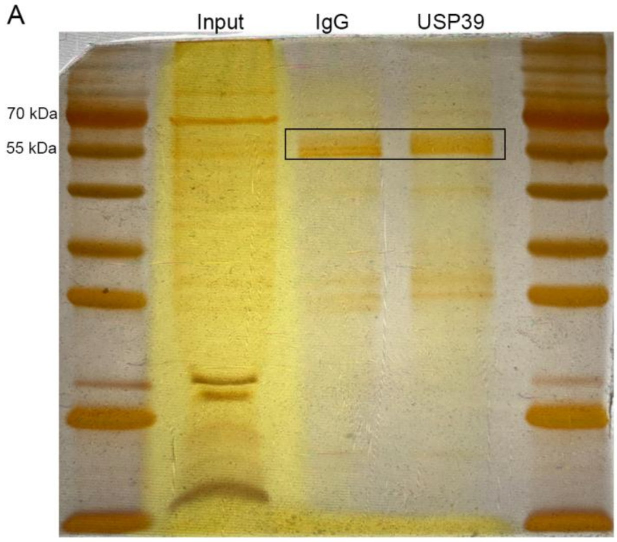

The image of sliver staining to identify the interacting proteins. (A) Co-IP sliver staining assay and spectrometry analysis assessed the potential proteins combined with USP39.

Supplement Figure 8.

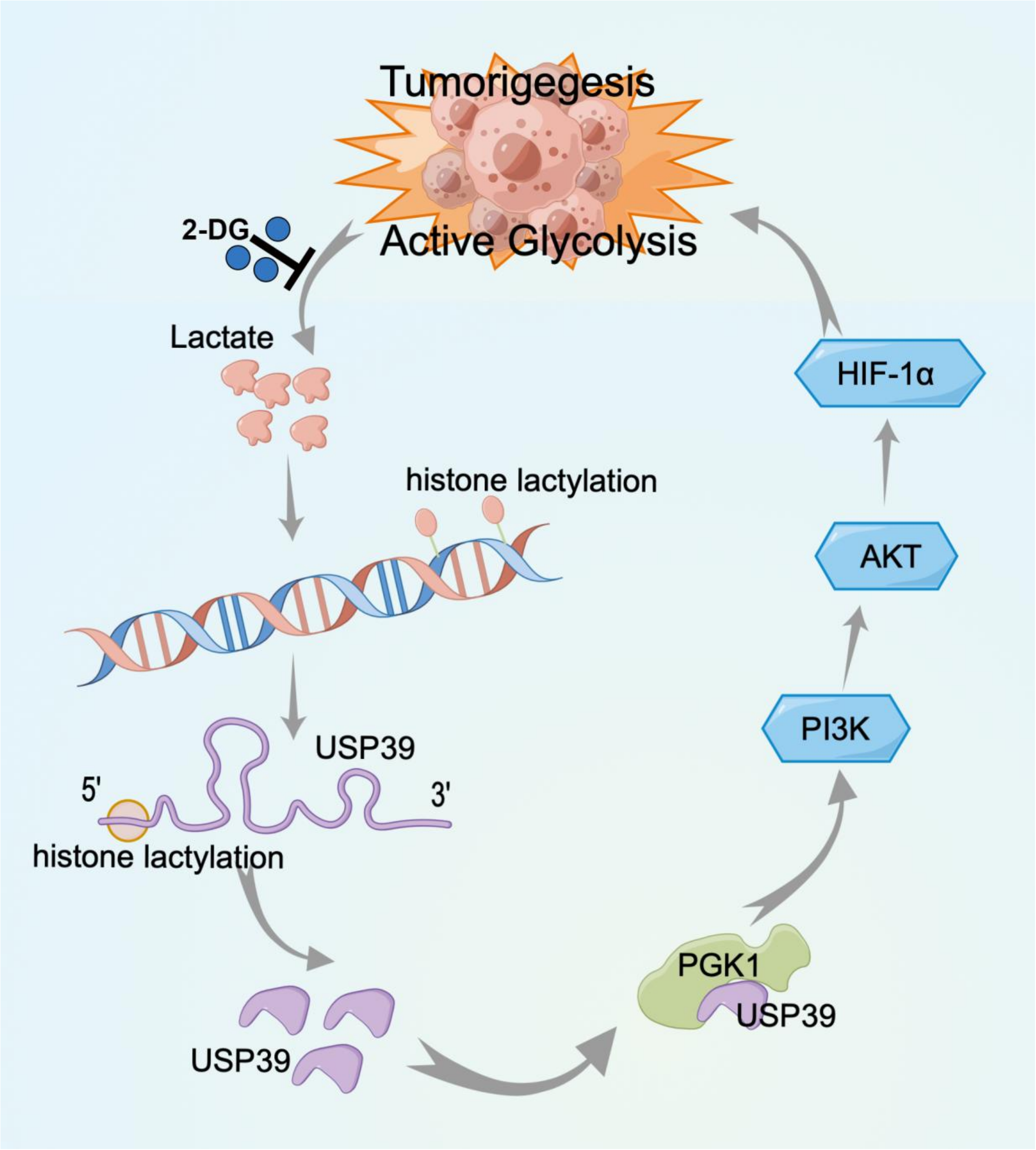

In endometrial carcinoma cells, a higher histone lactylation level induced by active glycolysis promotes the transcription of USP39, which interacts with PGK1 to activate the PI3K/AKT/HIF-1α signaling pathway, and contributes to the malignant progression of endometrial carcinoma. This image was painted by Figdraw (www.figdraw.com).
