# Supplementary figures and images for: Histone lactylation promotes malignant progression by facilitating USP39 expression to target PI3K/AKT/HIF-1α signal pathway in endometrial carcinoma
Source: Cell Death Discov. 2024 Mar 8;10:121. doi: 10.1038/s41420-024-01898-4 (PMC10923933; doi:10.1038/s41420-024-01898-4)

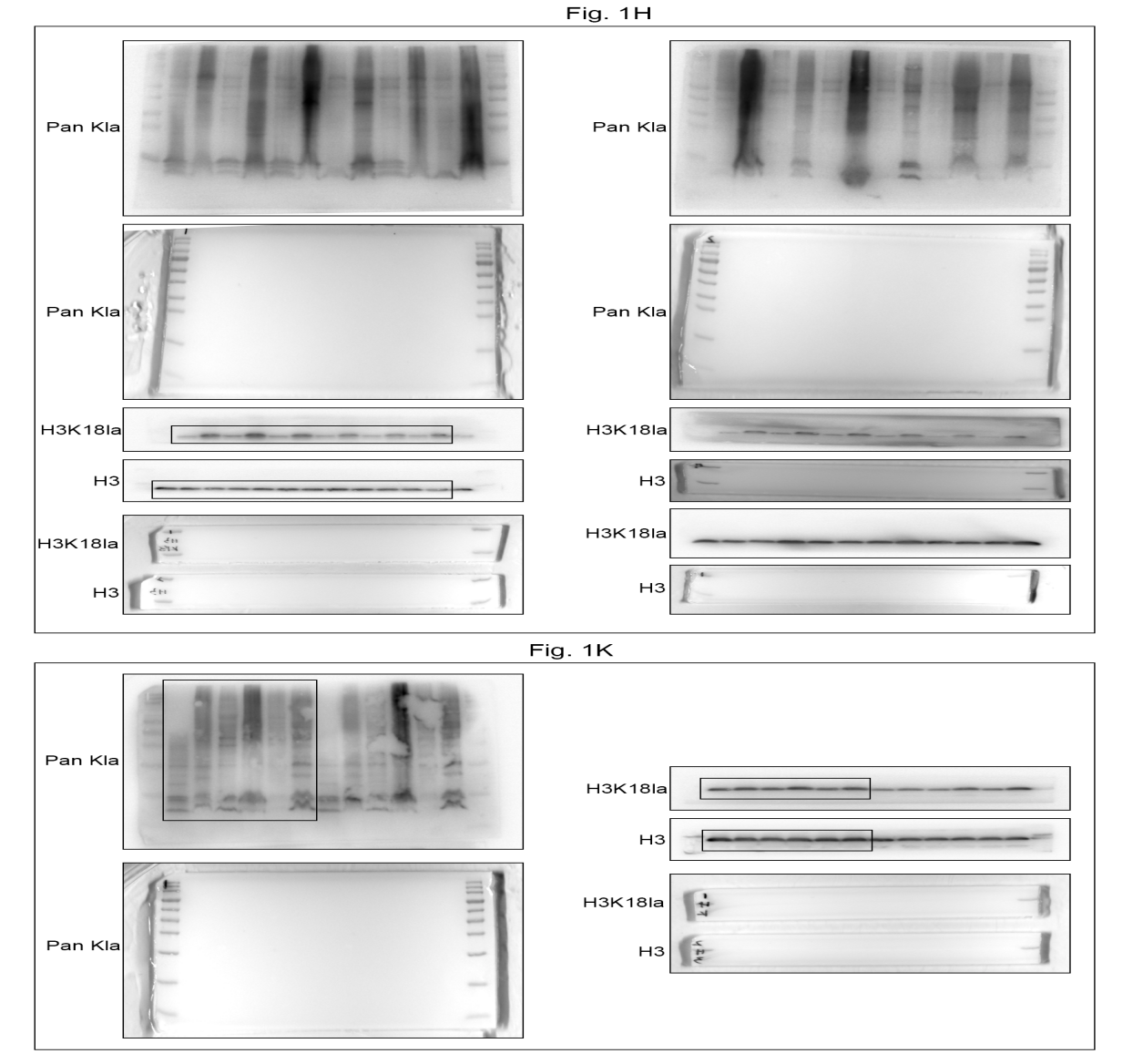


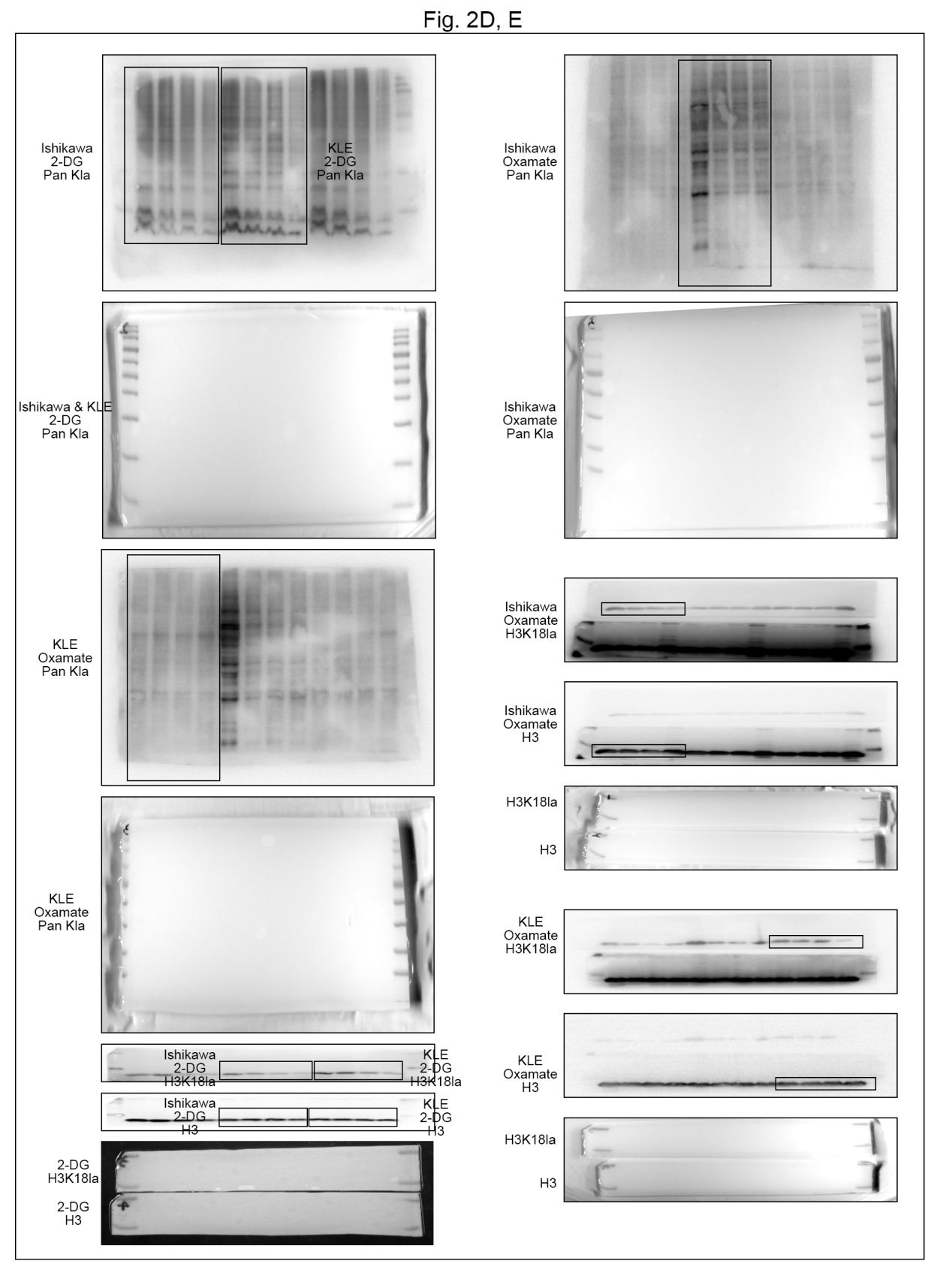


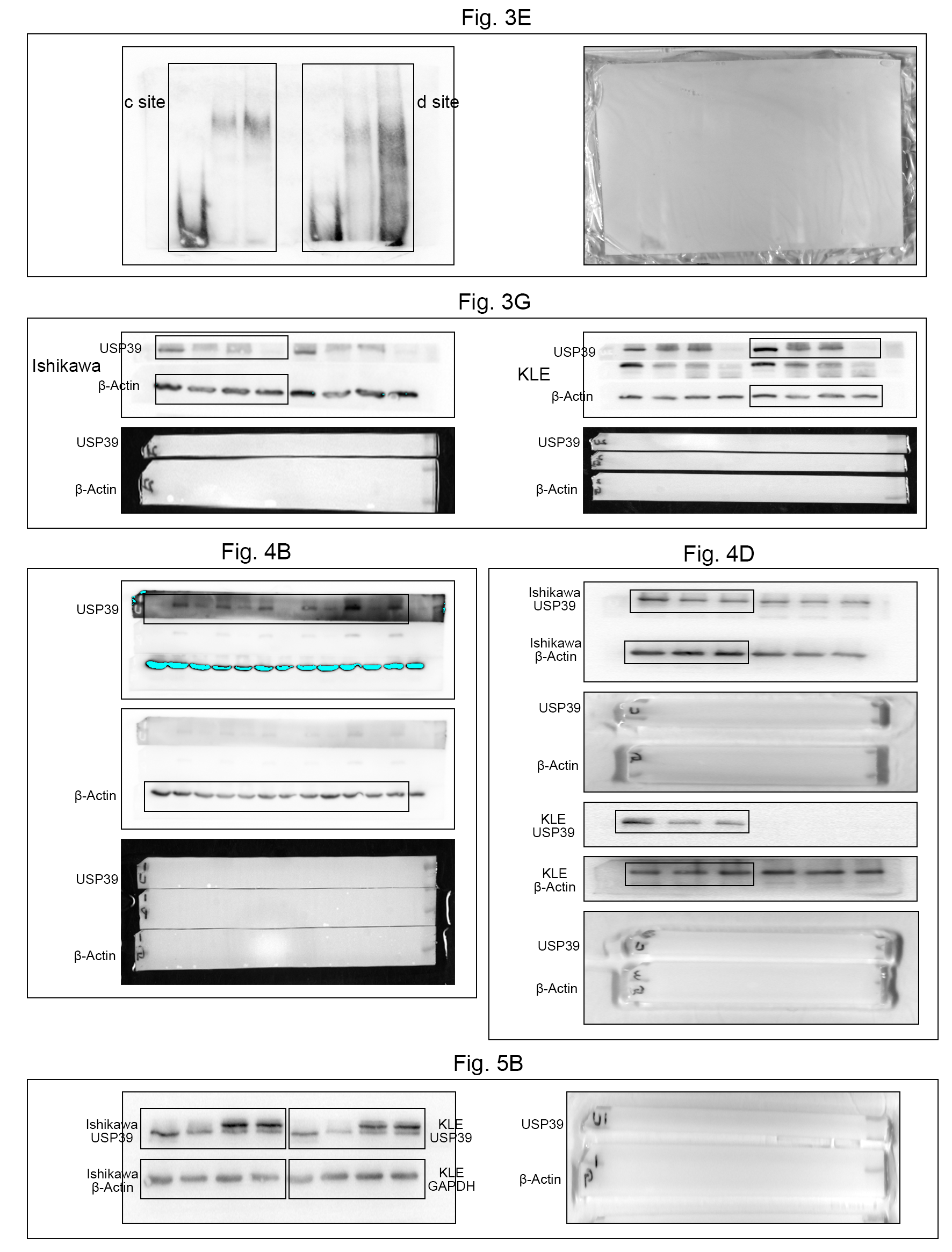


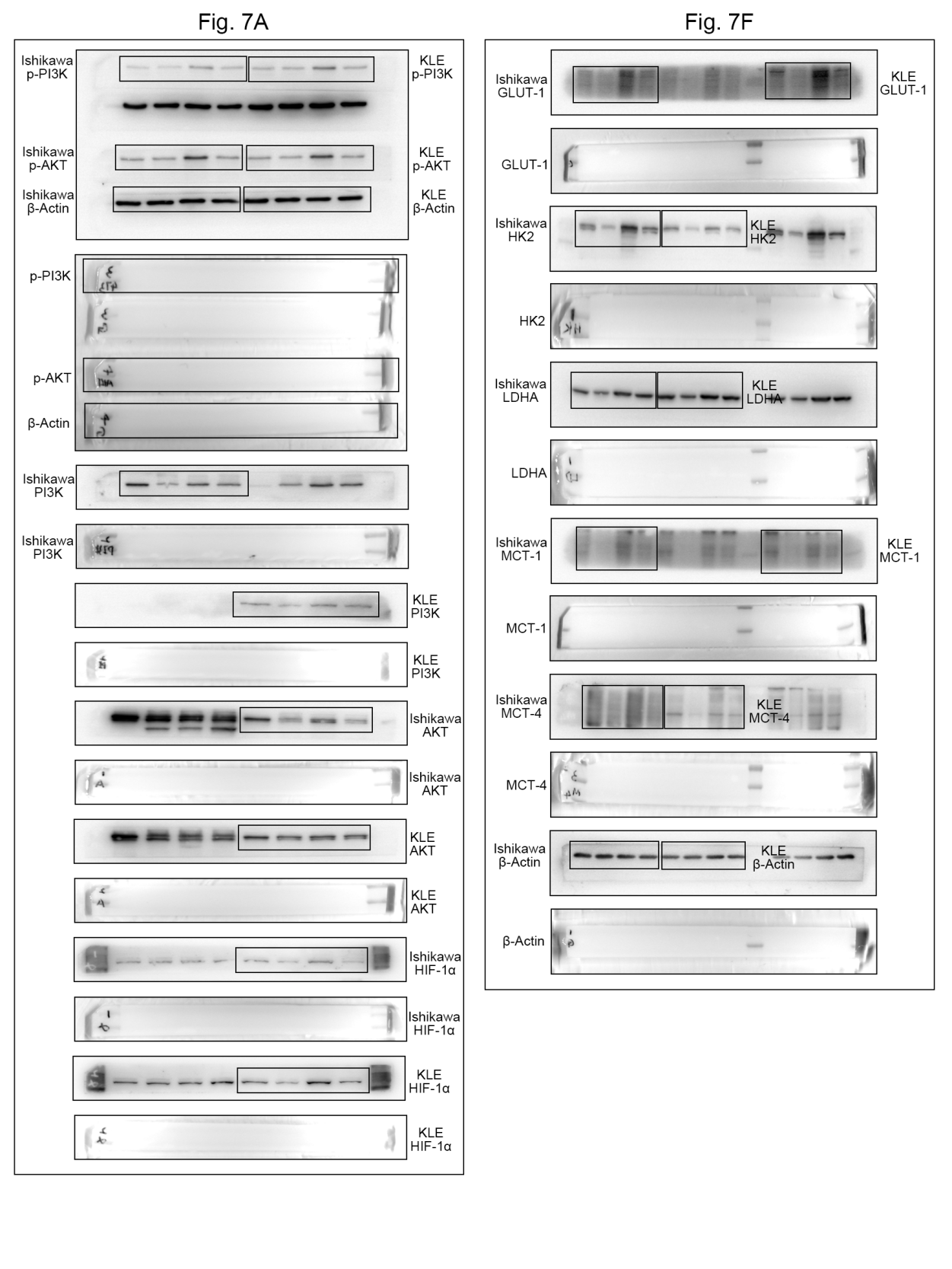


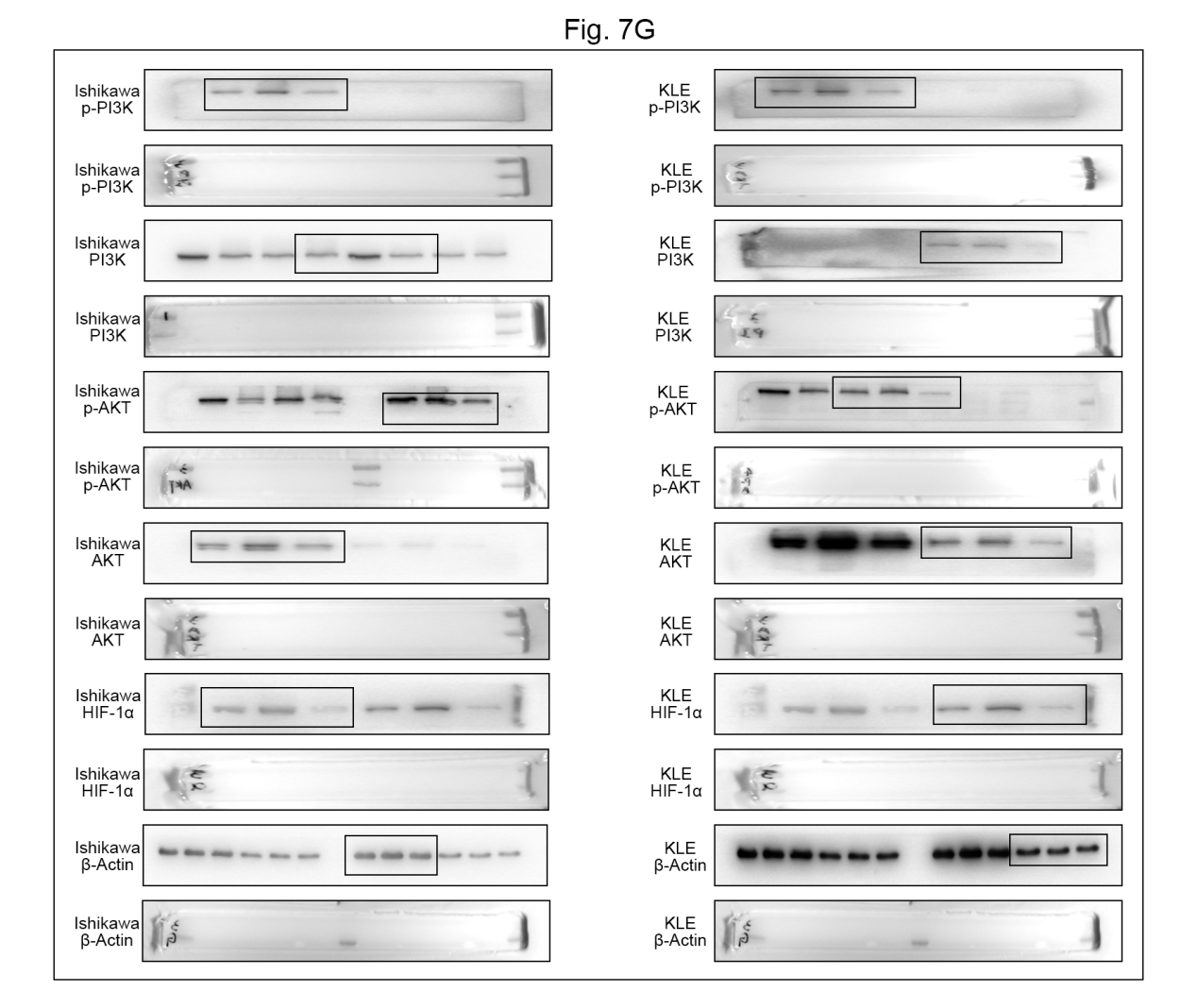


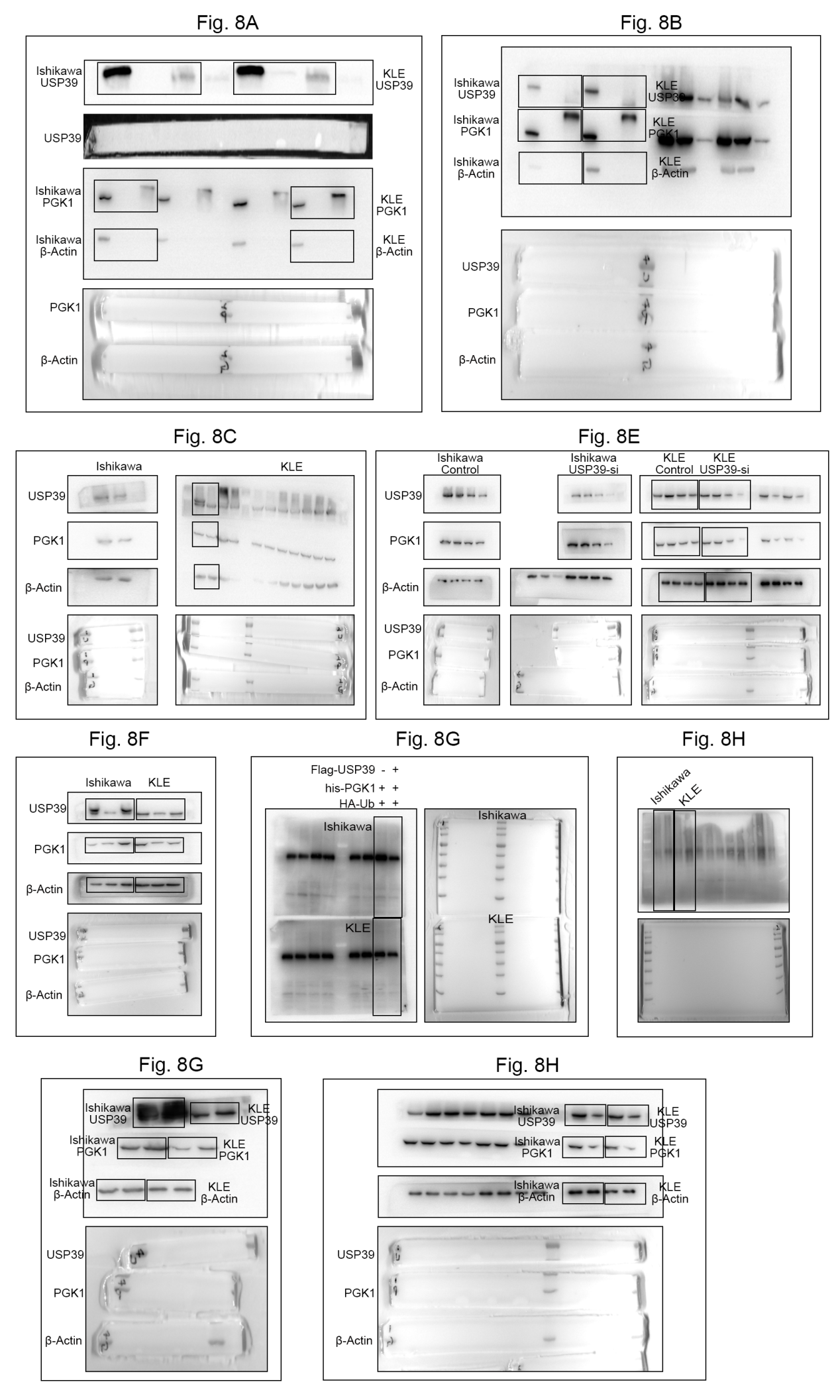


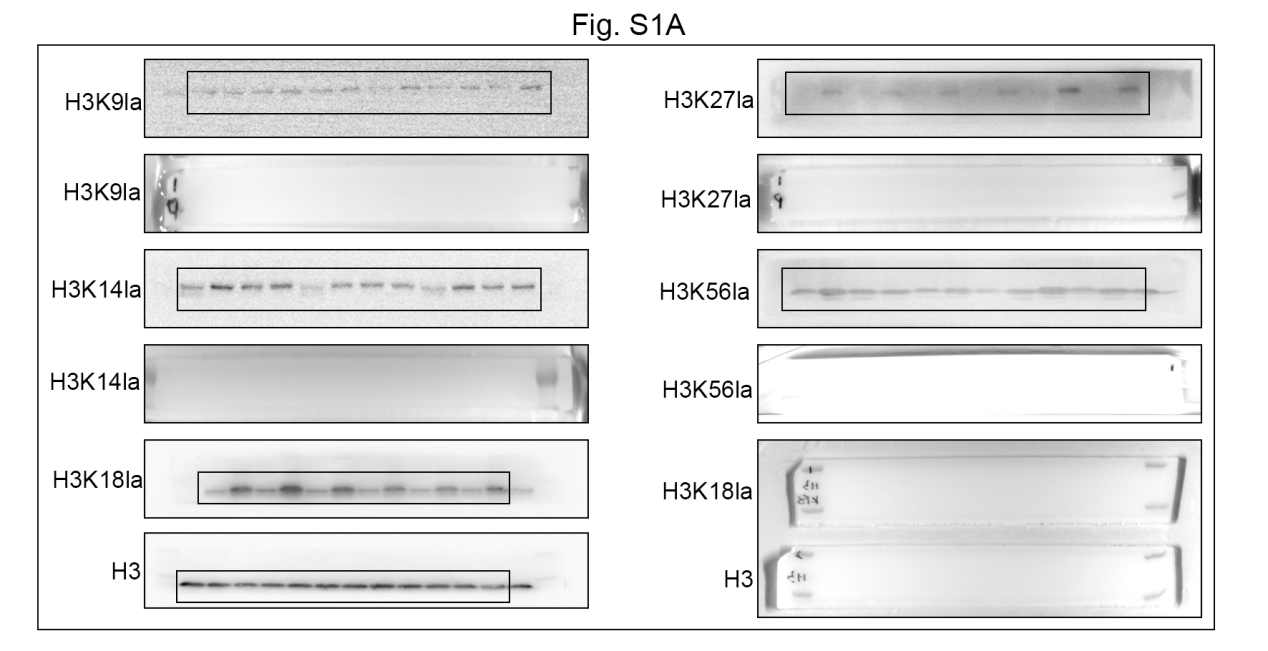


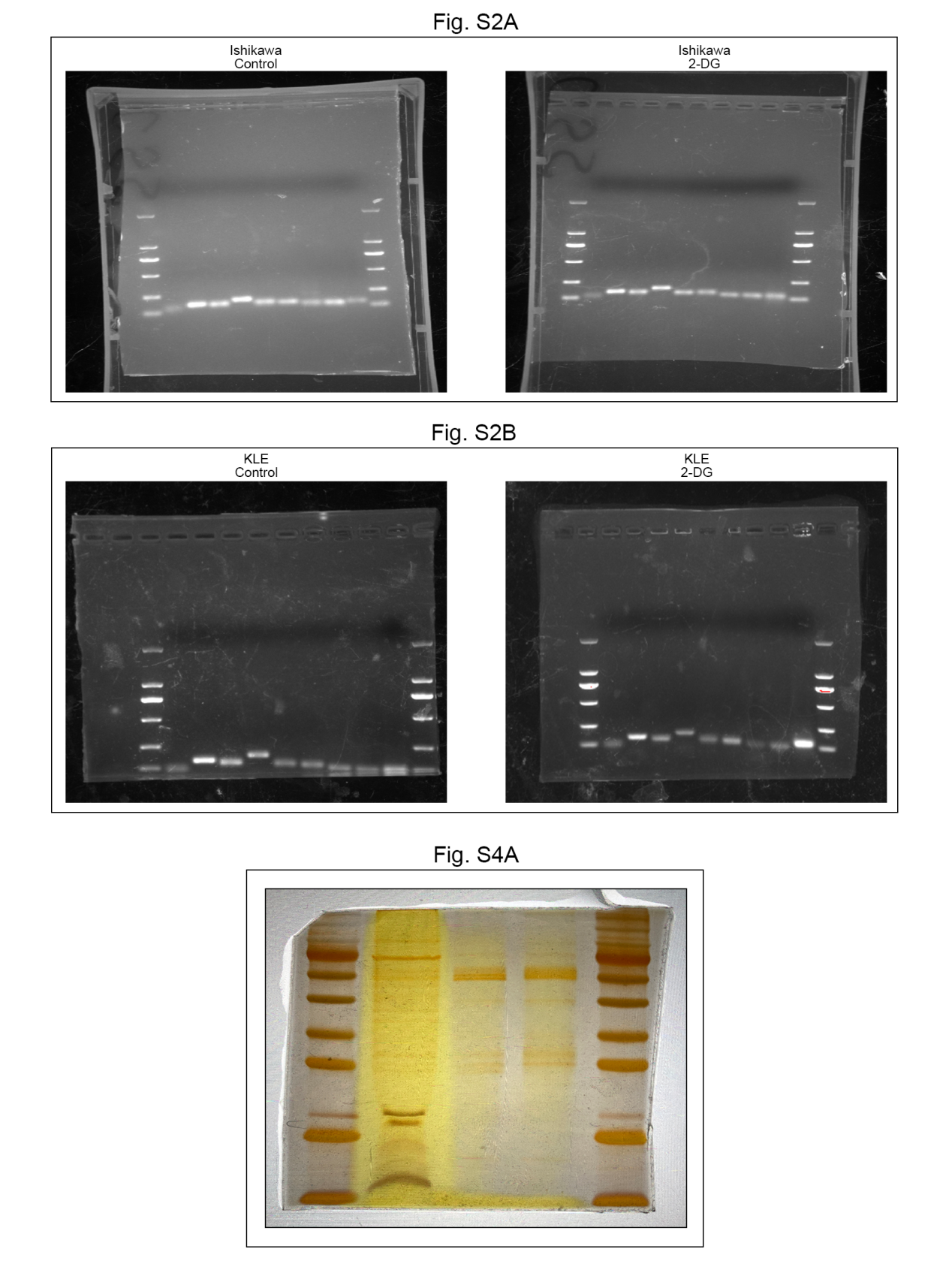

Supplement: Supplementary file 4 — Additional file 4 Uncropped western blotting analysis. [file 41420_2024_1898_MOESM4_ESM.docx]
